# Physical activity dynamically moderates the impact of multimorbidity on the trajectory of healthy aging over sixteen years

**DOI:** 10.1186/s12877-024-05067-1

**Published:** 2024-06-28

**Authors:** Nnaelue Godfrey Ojijieme, Tieying Feng, Chin Man Chui, Xinzhu Qi, Yuan Liu

**Affiliations:** 1 School of Public Policy and Administration, Xi’an Jiaotong University, 28 Xingqing Road, Xi’an, Shaanxi 710049 China; 2 School of Business, Macau University of Science and Technology, Taipa, Macau 999078 China; 3 Institute of Development Economics, Macau University of Science and Technology, Taipa, China

## Abstract

**Background:**

Research examining the healthy aging trajectory of retired older adults with multimorbidity is limited, leaving uncertainties regarding the optimal physical activity (PA) intensity and frequency necessary to sustain healthy aging during retirement.

**Methods:**

Our study investigated the moderating effects of PA on the healthy aging trajectories of retired older adults living with multimorbidity in the United States (US). We utilized data from 1,238 retired individuals aged 50 to 102 who contributed 11,142 observations over 16 years from the Health and Retirement Study (HRS). We employed mixed effects modeling to assess the impact of various classes of multimorbidity on this group and examine how different PA, PA intensities, and PA frequencies influence the disability, physical, and cognitive functioning domains of healthy aging.

**Results:**

The results reveal that while outcomes differed significantly, retired older adults in the US attained healthy aging at baseline. However, their ability to maintain healthy aging declined over time, with multimorbidity, especially musculoskeletal and neurological conditions, accelerating this decline. Fortunately, PA, especially light to moderate intensities, is associated with improving healthy aging and moderating the impact of multimorbidity on the disability and cognitive functioning domains of healthy aging. However, the specific moderating effects of PA depend on its frequency, intensity, and chronic conditions.

**Conclusions:**

The significant variability in healthy aging attainment among retired older adults underlies the need to consider these differences when addressing healthy aging issues in the US. Accounting for these variations would aid in evaluating the potential impact of future interventions and contribute to achieving health equity. Fortunately, our dynamic findings facilitate this objective by identifying specific frequencies and intensities of PA tailored to different aspects of multimorbidity and healthy aging. This highlights PA, especially light-to-moderate intensity, as an essential, cost-effective, and amenable strategy for alleviating the impact of multimorbidity on healthy aging.

**Supplementary Information:**

The online version contains supplementary material available at 10.1186/s12877-024-05067-1.

## Background

Healthy aging encompasses developing and maintaining sufficient functioning that enhances older adults’ independent living, well-being, and life satisfaction [1]. It is especially relevant for the retired population who face unique post-retirement challenges, such as increased sedentary lifestyles that exacerbate disabilities and functional decline [2]. Healthy aging attainment is also essential in the United States (US) because the rapidly expanding older adult population fuels concerns about health expenses and access to affordable care. Although more than half of US older adults express worries about high post-retirement health care expenses, and some others express a preference for death over moving into the mostly expensive nursing homes [3, 4], they may only be able to avoid these health expenses and nursing homes if they can minimize disability and maintain sufficient functional independence over time. Despite abundant research investigating the disability and functioning domains of healthy aging and their predictive factors in a cross-sectional context, there remains a significant gap in our understanding of the simultaneous long-term impact of risk factors and cost-effective interventions such as multimorbidity and physical activities on healthy aging [5–7].

Multimorbidity, the ‘co-occurrence of multiple chronic conditions in a single individual,’ is among the most significant healthy aging risk factors affecting over 80% of the US older adult population [8–10]Multimorbidity creates a vicious cycle of pain, depression, and functional limitations, which, in turn, exacerbates existing chronic conditions and healthy aging decline. Given its chronic nature, multimorbidity impoverishes US older adults through regular medical care, with medical expenses due to multimorbidity accounting for more than 70% of the total health expenditure of this subpopulation [8–12]. Therefore, investigating cost-effective and readily amenable long-term strategies is critical to ameliorating the impact of multimorbidity on the healthy aging of retired US older adults.

One such strategy is physical activities (PA), which entails deliberate engagement in events involving bodily movements and energy expenditure [13]. PA is ideal for healthy aging because it can be freely accessible, and its intensity is more adjustable to individual needs and abilities. PA helps alleviate chronic disease risks, disability, functional decline and enhances long-term health [14–16]. Notwithstanding its well-established benefits, about half of all US adults do not engage in PA [14]. Perhaps this low engagement stems from the fact that despite enormous PA guides, there is limited evidence on how retired older adults with multimorbidity can safely engage in PA, including which PA or intensity is ideal for specific chronic conditions [17, 18]. This study addresses these concerns by investigating the moderating impact of different physical activities and intensities on the association between healthy aging and the different classes of chronic conditions constituting multimorbidity.

## Healthy aging constructs

Consensus on healthy aging constituents remains elusive, with a stem of scholars exploring individual healthy aging domains that include disability [15], physical [5], social [19], and cognitive functioning [7]. In contrast, others employ Rowe and Kahn’s successful aging constructs that amalgamate the ‘absence of diseases’ to the domains above [20] or take refuge in the healthy aging index [21]. Other schools opine that while individual measures incomprehensively assess complex aging dynamics, the core of Rowe and Kahn’s conception is too restrictive and can underestimate healthy aging [22, 23]because its’ absence of diseases’ criterion flags over 90% of the US older adults as unhealthy, contradicting the high US health-adjusted life expectancy [22, 23]. The healthy aging index, on the other hand, concatenates numerous variables that complicate meaningful interpretation and inference.

By continuously relaxing Rowe and Kahn’s successful aging model from its most (level 1) to least (level 3) restrictive contexts, McLaughlin et al. find that the model excluding the ‘absence of diseases’ domain (level 3) is more reflective of current societal realities. For instance, while the ‘level 1’ (core Rowe and Kahn’s model) measure found that only 3% of the US’s older adults attained healthy aging, the ‘level 3’ measure indicated that a more realistic 30% achieved healthy aging. According to their perspective, older adults can attain healthy aging if diseases do not hinder their abilities and independent functioning; hence, the absence of disease is an insufficient condition for healthy aging. Besides, technological advances since the formation of Rowe and Kahn’s model have ensured that older adults live longer despite disease prevalence [24]. The exclusion of the disease domain is where some scholars argue that although similar, healthy aging differs from successful aging [25].

Therefore, in alignment with São José et al.‘s perspective that sustainable healthy aging indicators should assess one’s capability rather than a mere focus on achievement (as in the level 1 construct) [26], we infer that the augmented Rowe and Kahn’s model (depicting the disability, physical, and cognitive functioning) offers a more nuanced healthy aging assessment. We also chose the augmented measure because we believe that the high prevalence of multimorbidity in the US would make assessing healthy aging with the disease absence criterion inadequate. Furthermore, independently assessing these three domains is ideal for understanding how the interaction between multimorbidity and physical activities or intensities influences each domain in the overall aging process. The room for this in-depth analysis is limited if we conduct our analysis with healthy aging measures utilizing a single variable or index. The level 3 construct also reasonably estimated the prevalence of healthy aging in China [27].

## Hypothesis development

### Healthy aging trajectory

While healthy aging measures older adults’ complex aging dynamics, especially their abilities and functioning, it is well understood that individuals’ ability to maintain these functions naturally declines with age [5, 21]. Thus:

#### Hypothesis 1

*The retired older adults’ healthy aging attainment in the disability, physical, and cognitive functioning domains declines over time by depicting negative trajectory slopes*.

### Multimorbidity and healthy aging

Although healthy aging naturally declines over time, multimorbidity has been found to exacerbate the decline rate of healthy aging over time [5, 6]. Though this steeper decline in older adult’s ability to maintain independent functioning is a global phenomenon [10], multimorbidity is equally a severe concern in the US, with a prevalence of 80%, while accounting for approximately 70% of total health expenditure of the older adults’ population [28]. Thus:

#### Hypothesis 2

*Multimorbidity exacerbates the decline of healthy aging in the disability, physical, and cognitive functioning domains by reducing their baseline attainment and accelerating their rate of decline*.

### Physical activity and healthy aging

Because multimorbidity is expensive and challenging to eliminate, more scholars are exploring sustainable behavioral interventions such as PA because it is primarily free, amenable, and more accessible to retired older adults than other factors that are rigid to change at old age [15, 29]. PA improves older adults’ balance disorders and greatly diminishes their fall probability [30]Gow et al. also reported its immediate and lasting effects [31], observing that older adults with higher PA levels experience significantly better cognition at baseline and follow-up [31]. Similarly, Middleton et al. observed that while engaging in PA throughout a lifetime significantly reduced cognitive impairment odds in old age, those who engaged in any PA at any stage of their life had lower cognitive impairment odds relative to those without any PA [32]. Hence, we expect PA to enhance healthy aging by slowing the decline rate of healthy aging. Thus:

#### Hypothesis 3

*Older adults who engage in physical activities are expected to have higher baseline healthy aging in the domains of disability, physical, and cognitive functioning as they age, with a slower decline in these domains than their counterparts with no physical activities*.

### The moderating role of physical activities on the association between healthy aging and multimorbidity

Since older adult’s ability to achieve healthy aging declines over time, multimorbidity exacerbates healthy aging decline, and physical activity improves healthy aging; thus:

#### Hypothesis 4

*Older adults with multimorbidity who engage in physical activity will have higher baseline healthy aging and experience slower declines than those who suffer from multimorbidity but do not engage in physical activities.*

Following the four hypotheses stated above, this study aims to contribute to the ongoing healthy aging discourse by investigating how different physical activities and intensities moderate the healthy aging (in the disability, physical, and cognitive functioning domains) of retired US older adults with multimorbidity. Unlike previous studies that did not make this distinction [21, 22, 27, 29], we focus on the retired population because we can more easily ascertain the impact of PA on their healthy aging. After all, they are more likely to live a sedentary lifestyle relative to the subpopulation that could engage in other forms of physical activities through their exposure to employment.

We make several contributions to the healthy aging discourse: First, we used the most recent longitudinal study in the US that followed participants for 16 years, sufficiently capturing life-course changes. Second, using the latest US data, we establish how the counteractive effects of PA and multimorbidity simultaneously influence the levels and rate of change of healthy aging in the disability, physical, and cognitive functioning domains. Third, we identify the specific PA and PA intensities that retired older adults must maintain based on the kinds of chronic conditions they suffer. Our findings will provide extrapolatable evidence to guide retired older adults living with various classes of multimorbidity on the specific PA and intensities to attain more favorable healthy aging in the domains explored in this study [33], which would be essential for furthering our understanding of healthy aging development and influencing aging policies.

## Methods

### Participants and procedures

This study utilized data from the latest Health and Retirement Study (HRS), conducted every two years from 1992 to 2020. The HRS is a nationally representative survey that captures the sociodemographic data of middle-aged and retired older adults from approximately 23,000 US households and is supported by the National Institute on Aging (NIA U01AG009740) [34]. Similar to studies evaluating the retired population [35], we e only consider the individuals who are “completely retired” between 2004 and 2020. Following this screening, data missingness issues were significantly mitigated as data from the remaining 1238 individuals (aged 50 to 104) were mostly nonmissing. Note that the availability of physical activity data prompted the choice of 2004 as the base period for this study (See Table 1 for the complete descriptive statistics breakdown).

### Measures

#### Healthy aging

Based on the augmented Rowe and Kahn’s model (i.e., Mclaughlin et al.‘s level 3 construct), we assess the disability, physical, and cognitive functioning domains of healthy aging. The disability domain estimates retired older adults’ ability to independently perform ten activities of daily living (ADL) and the instrumental activities of daily (IADL): “bathing, eating, dressing, walking across a room, and getting in or out of bed, using a telephone, taking medication, handling money, shopping, and preparing meals.” We reverse-coded and summed the composite scores of these ten items such that they range from 0 = ‘inability to perform all tasks’ to 10 = ‘ability in all tasks.’ Deviating slightly from the parent model’s prescription (suggesting the maximum score for this domain), we believe it is reasonable to classify individuals with a disability domain score ≥ 9 as having ‘sufficient ability’ since approximately 0% of the sample scored 10 [22, 27, 36].

The physical functioning domain assesses retired older adults’ capacity to engage in nine activities requiring ‘mobility’ and the use of ‘large muscles’: “walking several blocks, walking one block, walking across the room, climbing several flights of stairs, climbing one flight of stairs, sitting for two hours, getting up from a chair, stooping or kneeling or crouching, and pushing or pulling a large object.” We reverse-coded and summed the composite scores of these items such that they range from 0 = ‘poor functioning’ to 9 = excellent functioning;’ with a physical functioning domain intercept ≥ 8 indicating high physical functioning [22, 27].

The cognitive functioning domain captures retired older adults’ overall cognitive health using the total cognition score. The total cognition score is an ordinal outcome (ranging from 0 to 35) and is the sum of the weighted composite scores of the total recall and mental status indices — word recall (range 0—20); serial 7 subtraction (range 0—5); backward counting (range 0—2); objects (range 0—2), date (range 0—4), and president/vice president recognition (range 0—2). A cognition score tending to 35 entails excellent cognition in all cognition indices. A cognitive domain intercept ≥ 22 indicates high cognitive functioning [22, 37, 38].

#### Multimorbidity

study participants reported doctor’s diagnoses of the following eight chronic conditions: “1) high blood pressure or hypertension; 2) diabetes or high blood sugar; 3) cancer or a malignant tumor of any kind except skin cancer; 4) chronic lung disease except asthma such as chronic bronchitis or emphysema; 5) heart attack, coronary heart disease, angina, congestive heart failure, or other heart problems; 6) stroke or transient ischemic attack (TIA); 7) emotional, nervous, or psychiatric problems; and 8) arthritis or rheumatism.” Based on relevant research, we grouped these conditions into five different multimorbidity classes as follows: *cardiometabolic* (diabetes, heart problems), *neurological* (hypertension, stroke, and psychiatric problems), *musculoskeletal* (arthritis), *respiratory* (chronic lung disease), and *cancer*. Each class is coded 0 (no chronic condition) and 1 (at least one chronic condition) and entered as distinct variables to ascertain the effects of each class on healthy aging. This approach is appropriate because although all classes of multimorbidity predict healthy aging, there is a low probability of correlation amongst them since having one class of chronic condition does not necessarily influence the occurrence of another in one individual [39, 40].

#### Physical activity and intensity

retired older adults reported their ‘*frequency’* of engaging in light, moderate, and vigorous physical activity intensities. *Light physical activities* include “vacuuming, home repairs, sports, or other mildly energetic acts.” *Moderate physical activities* include “gardening, walking, dancing, stretching, and cleaning the car.” *Vigorous physical activities* included “running or jogging, cycling, playing tennis, digging, gym workouts, and swimming.” The frequency of each physical activity intensity was reverse-coded and ranged from 1 = none, 2 = one to three days per month, 3 = one day per week, 4 = more than one day per week, and 5 = daily physical activities.

Furthermore, we included established healthy aging covariates such as age, sex, race, marital status, educational attainment, socioeconomic status, body mass index (BMI), smoking and drinking status, and wave to control for retired older adults’ sociodemographic characteristics [15, 22]. Age is entered as a continuous variable ranging from 50 to 104 years old. We coded sex as 1 = female and 2 = male. Race ranged from 1 = white/Caucasian, 2 = black/African American, 3 = other. We coded marital status as 1 = “married, married (spouse absent), partnered, and 0 = separated, divorced, widowed, and never married, separated, and divorced.” Educational attainment is entered as 1 = less than high school, 2 = GED, 3 = high school graduate, 4 = some college, 5 = college and above. Based on Amadeo et al., socioeconomic status is calculated as the sum of total assets (less debt) and is coded as (-141,999 to 6030 = 0) poverty class, (6030.1 to 42,000 = 1) lower-middle class, (42,001 to 104,000 = 2) middle class, (104,001 to 201,000 = 3) upper middle class, (201,001 to 608,000 = 4) wealthy, and (608,001 and above = 5) super wealthy [41]. BMI is a continuous variable ranging from 12.6 to 58.6. Smoking status = 0 for non-smokers and 1 for smokers. Drinking frequency ranged from 0 = none to 7 = daily drinking. We included wave and wave^2^ to account for the influence of repeated measurements taken on individuals over different waves and for retired older adults’ healthy aging status depending on time [42].

### Analysis

This study employed the mixed effects model with an unstructured covariance matrix and random intercept to estimate the longitudinal association between multimorbidity, physical activities, and the disability, physical, and cognitive functioning domains of healthy aging [43]. The mixed effects model is a multilevel statistical technique utilizing fixed and random effects to analyze nested data [43–45]This method is beneficial for analyzing variations within (fixed effects) and between (random effects) individuals over time and has been widely applied in the literature [46, 47]. Hence, we used the individuals’ ID and wave to construct nine mixed effects models. Each healthy aging domain was constructed three times, with each model assessing the interaction between the frequency of PA intensities and the five multimorbidity classes. Specifically, we introduced the three different PA intensities separately for each healthy aging domain model and interacted with them the multimorbidity classes of cancer, cardiometabolic, neurological, musculoskeletal, and respiratory conditions. We specify the base models for the three PA intensities below:

1 $$\begin{aligned} H{A_{dit}}\, = \,{\pi _{0i}}\, + \,{\beta _{1 - 5}}M{M_{it}}\, + & \\ & {\beta _6}PA{L_{fit}}\, + \,{\beta _{7 - 31}}{\text{(}}M{M_{it}} \times PA{L_{fit}}{\text{)}} \\ & {\beta _{32 - 37}}TV{C_{it}}\, + \,{\beta _{38 - 40}}TI{C_i} \\ & {\beta _{41 - 49}}(WAV{E_t} \times WAV{E_t})\, + \,I{D_i} \\ & WAV{E_t}\, + \,WAV{E^2}_t\, + \,{ \in _{it}} \\ \end{aligned}$$

2 $$\begin{aligned} H{A_{dit}}\, = \,{\pi _{0i}}\, + \,{\beta _{1 - 5}}M{M_{it}}\, + & \\ & {\beta _6}PA{M_{fit}}\, + \,{\beta _{7 - 31}}{\text{(}}M{M_{it}} \times PA{M_{fit}}{\text{)}} \\ & {\beta _{32 - 37}}TV{C_{it}}\, + \,{\beta _{38 - 40}}TI{C_i} \\ & {\beta _{41 - 49}}(WAV{E_t} \times WAV{E_t})\, + \,I{D_i} \\ & WAV{E_t}\, + \,WAV{E^2}_t\, + \,{ \in _{it}} \\ \end{aligned}$$

3 $$\begin{aligned} H{A_{dit}}\, = \,{\pi _{0i}}\, + \,{\beta _{1 - 5}}M{M_{it}}\, + & \\ & {\beta _6}PA{V_{fit}}\, + \,{\beta _{7 - 31}}{\text{(}}M{M_{it}} \times PA{V_{fit}}{\text{)}} \\ & {\beta _{32 - 37}}TV{C_{it}}\, + \,{\beta _{38 - 40}}TI{C_i} \\ & {\beta _{41 - 49}}(WAV{E_t} \times WAV{E_t})\, + \,I{D_i} \\ & WAV{E_t}\, + \,WAV{E^2}_t\, + \,{ \in _{it}} \\ \end{aligned}$$

Where: *i* represents the retired older adults in this study from individual 1 to 1,238. And *t* represents the sixteen waves of the HRS utilized for this study from years 2004 to 2020.

The dependent variable (*HA*_*dit*_) represents the disability, cognitive and physical functioining healthy aging domains (*d*) for individual *i* in wave *t*. *MM*_*it*_ represents the five multimorbidity classes of cancer, cardiometabolic, musculosckeletal, neurological, and respiratory conditions for individual *i* in wave *t*. *PAL*_*fit*_ represents the frequency (*f)* of engaging in light PA intensities for individual *i* in wave *t. PAM*_*fit*_ represents the frequency (*f)* of engaging in moderate PA intensities for individual *i* in wave *t. PAV*_*fit*_ represents the frequency (*f)* of engaging in vigorous PA intensities for individual *i* in wave *t. TVC*_*it*_ represents the time-varying covariates (age, SES, BMI, drinking and smoking status) for individual *i* in wave *t*. *TIC*_*it*_ captures the time-invariant covariates (race, sex, and marital status) for individual *i*. Where: *π*_*0i*​_ = *β*_0_ + µ_*0i*​_ represents the individual-level healthy aging intercept and random effects. ID is the random effect term for individual *i*, representing the deviation of individual i from the population mean. *WAVE*_*t*_ and *WAVE*^2^_*t*_ is the random effect term for time and point t, representing the deviation of time point t from the overall time trend considering that changes across time could be nonlinear. ${ \in _{it}}$ is the residual error term, representing the difference between the observed and predicted values of healthy aging.

### Data management

After reshaping the data of the 1,238 individuals to its long format, the observations for the various models fell short of the supposed 11,142 total. Assuming that data are ‘missing at random,’ we invoked the Multivariate Imputation by Chained Equations (MICE) technique to address data missingness issues for the nine mixed effects models [48, 49]. Afterward, we verified that the sample statistics of the imputed data were quantitatively similar to that of the original data (See Tables S1 to S3 in the Additional File). Furthermore, we conducted sensitivity analysis by performing a complete case analysis for the nine models in this study. Therefore, obtaining similar results would bolster the robustness of our findings. All analysis were performed using STATA version 18 [50].

## Results

An abridged version of this study’s sample capturing the baseline to terminal period characteristics is reported in Table 1 below (see the complete version in the Table S7 of the Additional File). It communicates a negative relationship between the three healthy aging domains and time. For instance, while average cognitive and physical functioning declined between the base and terminal periods, more retired older adults reported having less indepence due to disability in the same timeline. The same trend is observed for all physical activity intensities, indicating that retired older adults are increasingly unable to maintain xonstant levels of physical activities over time. Conversely, more retired older adults report being diagnosed with chronic conditions for all the multimorbidity classes over time. Unsurprisingly, the prevalence of mutimorbidity is more apparent when we reconstruct the sample characteristics with respect to the number of chronic conditions retired older adults suffer (See Table S8 in the Additional File). Furthermore, at baseline, most of the study participants are white (85.3%), female (61.3%), high-school graduates (34.8%), married (72.4%), wealthy (35.3%), with an average age and BMI of 67 and 27.8, respectively.

**Table 1:** Sample characteristics

Variables	2004	Waves	2020	Total
		2018		
N	1,238 (11.1%)	1,238 (11.1%)	1,238 (11.1%)	11,142 (100.0%)
***Healthy Aging Domains***				
*Cognition Functioning*	23.865 (4.010)	21.310 (4.937)	20.628 (5.098)	22.444 (4.549)
*Disability*				
0	0 (0.0%)	4 (0.3%)	9 (0.7%)	17 (0.2%)
1	0 (0.0%)	7 (0.6%)	9 (0.7%)	22 (0.2%)
2	0 (0.0%)	7 (0.6%)	15 (1.2%)	39 (0.4%)
3	6 (0.5%)	15 (1.2%)	32 (2.6%)	79 (0.7%)
4	7 (0.6%)	24 (1.9%)	26 (2.1%)	104 (0.9%)
5	8 (0.6%)	48 (3.9%)	53 (4.3%)	218 (2.0%)
6	16 (1.3%)	52 (4.2%)	51 (4.1%)	268 (2.4%)
7	28 (2.3%)	72 (5.8%)	97 (7.8%)	465 (4.2%)
8	74 (6.0%)	146 (11.8%)	177 (14.3%)	1,143 (10.3%)
9	1,099 (88.8%)	863 (69.7%)	768 (62.0%)	8,776 (78.8%)
10	0 (0.0%)	0 (0.0%)	1 (0.1%)	2 (0.0%)
*Physical Functioning*				
0	7 (0.6%)	28 (2.3%)	38 (3.1%)	161 (1.4%)
1	19 (1.5%)	89 (7.2%)	89 (7.2%)	412 (3.7%)
2	28 (2.3%)	87 (7.0%)	95 (7.7%)	518 (4.7%)
3	61 (4.9%)	103 (8.3%)	96 (7.8%)	709 (6.4%)
4	75 (6.1%)	106 (8.6%)	119 (9.6%)	765 (6.9%)
5	79 (6.4%)	111 (9.0%)	122 (9.9%)	974 (8.7%)
6	113 (9.1%)	143 (11.6%)	119 (9.6%)	1,214 (10.9%)
7	172 (13.9%)	170 (13.7%)	151 (12.2%)	1,502 (13.5%)
8	248 (20.0%)	188 (15.2%)	171 (13.8%)	1,865 (16.8%)
9	436 (35.2%)	213 (17.2%)	238 (19.2%)	3,014 (27.1%)
***Multimorbidity***				
Cardiometabolic Conditions				
No	924 (74.6%)	547 (44.2%)	505 (40.8%)	6,432 (57.8%)
Yes	314 (25.4%)	691 (55.8%)	733 (59.2%)	4,702 (42.2%)
Neurological Conditions				
No	532 (43.0%)	237 (19.1%)	209 (16.9%)	3,108 (27.9%)
Yes	706 (57.0%)	1,001 (80.9%)	1,029 (83.1%)	8,026 (72.1%)
Musculoskeletal Conditions				
No	518 (41.8%)	265 (21.4%)	239 (19.3%)	3,332 (29.9%)
Yes	720 (58.2%)	973 (78.6%)	999 (80.7%)	7,802 (70.1%)
Respiratory Conditions				
No	1,169 (94.4%)	1,062 (85.8%)	1,045 (84.4%)	10,012 (89.9%)
Yes	69 (5.6%)	176 (14.2%)	193 (15.6%)	1,122 (10.1%)
Cancer				
No	1,074 (86.8%)	908 (73.3%)	879 (71.0%)	8,823 (79.2%)
Yes	164 (13.2%)	330 (26.7%)	359 (29.0%)	2,311 (20.8%)
***Light Physical Activity & Frequency***				
None	53 (4.3%)	225 (18.2%)	306 (24.8%)	1,240 (11.1%)
1—3 days per month	65 (5.3%)	108 (8.7%)	128 (10.4%)	867 (7.8%)
1 day per week	283 (22.9%)	350 (28.3%)	339 (27.5%)	3,013 (27.1%)
> 1 day per week	743 (60.0%)	433 (35.1%)	344 (27.9%)	5,020 (45.1%)
Everyday	94 (7.6%)	119 (9.6%)	116 (9.4%)	983 (8.8%)
***Moderate Physical Activity & Frequency***				
None	142 (11.5%)	392 (31.7%)	454 (36.8%)	2,403 (21.6%)
1—3 days per month	124 (10.0%)	124 (10.0%)	137 (11.1%)	1,163 (10.5%)
1 day per week	189 (15.3%)	161 (13.0%)	162 (13.1%)	1,771 (15.9%)
> 1 day per week	691 (55.9%)	414 (33.5%)	324 (26.3%)	4,683 (42.1%)
Everyday	90 (7.3%)	145 (11.7%)	156 (12.7%)	1,095 (9.9%)
***Vigorous Physical Activity & Frequency***				
None	639 (51.7%)	828 (67.0%)	897 (73.0%)	6,594 (59.4%)
1—3 days per month	116 (9.4%)	71 (5.7%)	70 (5.7%)	807 (7.0%)
1 day per week	110 (8.9%)	99 (8.0%)	70 (5.7%)	963 (8.7%)
> 1 day per week	340 (27.5%)	203 (16.4%)	154 (12.5)	2,448 (22.4%)
Everyday	32 (2.6%)	35 (2.8%)	38 (3.1%)	280 (2.5%)
***Race***				
White/Caucasian	1,056 (85.3%)	1,056 (85.3%)	1,056 (85.3%)	9,504 (85.3%)
Black/African American	154 (12.4%)	154 (12.4%)	154 (12.4%)	1,386 (12.4%)
Other	28 (2.3%)	28 (2.3%)	28 (2.3%)	252 (2.3%)
***Sex***				
Male	479 (38.7%)	479 (38.7%)	479 (38.7%)	4,311 (38.7%)
Female	759 (61.3%)	759 (61.3%)	759 (61.3%)	6,831 (61.3%)
***Education Attainment***				
Less than high school	160 (12.9%)	160 (12.9%)	160 (12.9%)	1,440 (12.9%)
GED	75 (6.1%)	75 (6.1%)	75 (6.1%)	675 (6.1%)
High-school graduate	431 (34.8%)	431 (34.8%)	431 (34.8%)	3,879 (34.8%)
Some college	292 (23.6%)	292 (23.6%)	292 (23.6%)	2,628 (23.6%)
College and above	280 (22.6%)	280 (22.6%)	280 (22.6%)	2,520 (22.6%)
***Marital status***				
Unmarried	341 (27.6%)	341 (27.6%)	341 (27.6%)	3,069 (27.6%)
Married	896 (72.4%)	896 (72.4%)	896 (72.4%)	8,064 (72.4%)
***Socioeconomic Status***				
Poverty class	69 (5.6%)	130 (10.5%)	136 (11.0%)	952 (8.6%)
Lower-middle class	87 (7.0%)	83 (6.7%)	92 (7.4%)	755 (6.8%)
Middle class	133 (10.7%)	116 (9.4%)	109 (8.8%)	1,070 (9.6%)
Upper middle class	178 (14.4%)	161 (13.0%)	147 (11.9%)	1,475 (13.2%)
Wealthy	437 (35.3%)	367 (29.6%)	369 (29.8%)	3,527 (31.7%)
Super wealthy	334 (27.0%)	381 (30.8%)	385 (31.1%)	3,355 (30.1%)
Age	67.222 (5.951)	81.303 (5.959)	83.32 (5.974)	75.292 (7.910)
Body Mass Index	27.767 (5.262)	27.614 (5.663)	27.178 (5.699)	27.909 (5.586)
Drinking status	1.324 (2.211)	1.156 (2.136)	1.061 (2.097)	1.226 (2.172)
Smoking status	0.093 (0.290)	0.041 (0.199)	0.038 (0.192)	0.061 (0.239)

*Note* Table 1 omitted characteristics from 2006 to 2016. The complete table is in the Additional File (Table S7). Cognitive functioning, age, drinking, body mass index, and smoking all report the means and standard deviations (in parentheses). The sample characteristics by the number of chronic conditions is reported in the Additional File (Table S8)

### The moderating effects of light physical activities intensities (and frequency)

Table 2 presents the mixed effects results for the moderating effects of light PA (and frequency) on the association between five multimorbidity classes and the physical functioning, and disability domains of healthy aging. It reports that engagement in light PA intensity is directly associated with improving healthy aging (across all domains), almost irrespective of the frequency (see the main effects portion of physical activity in Table 2). Conversely, retired older adults suffering neurological, musculoskeletal, and respiratory conditions are susceptible to worsening physical functioning (neurological: *β = -0.353*, *p* < 0.05; musculoskeletal: *β = -0.* 936, *p* < *0.01; respiratory:* β *= -0. 416,p* < 0.05) and disability (neurological: *β = -0.295,p* < 0.01;musculoskeletal: *β = -0.165,p* < 0.05; respiratory: β = -0.213, *p* < 0.05).

**Table 2:** The moderating effects of light physical activity intensity (and frequency) on healthy aging

	Cognitive Functioning	Physical Functioning	Disability
*Cardiometabolic Conditions*			
Yes	0.162	-0.040	-0.084
*Physical Activity (and Frequency)*			
1—3 days per month	0.803*	0.351*	0.319***
1 day per week	0.715*	0.654***	0.416***
> 1 day per week	0.539	0.629***	0.365***
Everyday	0.643	0.593***	0.318***
*Cardiometabolic Conditions # Physical Activity (and Frequency)*			
1—3 days per month	-0.206	-0.030	0.034
1 day per week	-0.037	-0.250**	-0.022
> 1 day per week	-0.247	-0.213*	0.026
Everyday	-0.272	-0.114	0.087
*Neurological Conditions*			
Yes	-0.149	-0.353**	-0.295***
*Neurological Conditions # Physical Activity (and Frequency)*			
1—3 days per month	-0.074	0.012	0.206**
1 day per week	-0.084	-0.064	0.176**
> 1 day per week	0.195	0.051	0.229***
Everyday	-0.125	0.081	0.274***
*Musculoskeletal Conditions*			
Yes	-0.157	-0.936***	-0.165**
*Musculoskeletal Conditions # Physical Activity (and Frequency)*			
1—3 days per month	-0.082	0.102	-0.013
1 day per week	0.049	0.144	0.071
> 1 day per week	0.476	0.236*	0.102
Everyday	0.761*	0.335**	0.125
*Respiratory*			
Yes	-0.327	-0.416**	-0.213**
*Respiratory Conditions # Physical Activity (and Frequency)*			
1—3 days per month	0.200	-0.164	0.273**
1 day per week	0.311	0.039	0.071
> 1 day per week	0.148	-0.165	0.006
Everyday	0.009	-0.378*	0.001
Cancer			
Yes	-0.264	-0.048	0.021
*Cancer # Physical Activity (and Frequency)*			
1—3 days per month	0.380	0.104	0.021
1 day per week	0.361	-0.029	-0.086
> 1 day per week	0.251	0.072	-0.052
Everyday	0.194	0.138	-0.072
*Race (Ref: White)*	-1.395***	-0.285***	-0.103**
*Sex (Ref: Male)*	0.837***	-0.561***	-0.061*
*Education Attainment (Ref: < High School)*	0.902***	0.179***	0.013
*Age*	-0.112***	0.015*	0.006**
*Marital Status (Ref: Unmarried)*	-0.116	0.139	0.019
*Socioeconomic Status (Ref: Low SES)*	0.508***	0.097***	0.038***
*Body Mass Index*	0.024*	-0.068***	-0.018***
*Drinking (Ref: No drinking)*	0.031	0.039***	0.011*
*Smoking (Ref: No smoking)*	-0.076	-0.059	-0.121**
*Wave (Ref: 2004)*			
2006	-0.136	-0.067	-0.026
2008	0.029	-0.143**	-0.025
2010	-0.531***	-0.288***	-0.063*
2012	-0.545***	-0.331***	-0.082**
2014	-0.682***	-0.574***	-0.212***
2016	-0.923***	-0.773***	-0.295***
2018	-1.032***	-1.139***	-0.497***
2020	-1.456***	-1.171***	-0.728***
Constant	25.844***	8.229***	8.654***
Variance (Wave)	-0.060	-0.556***	-1.124***
Variance (Wave^2)	-2.369***	-2.855***	-3.179***
Variance (Constant)	0.933***	0.518***	-0.583***
Covariance (Wave, Wave^2)	-1.601***	-1.538***	-1.536***
Covariance (Wave, Constant)	-0.557***	-0.478***	-0.734***
Covariance (Wave^2, Constant)	0.422***	0.322***	0.623***
Variance (Residuals)	1.044***	0.153***	-0.384***
Number of observations	11,142	11,142	11,142

***p < 0.01, **p < 0.05, *p < 0.1

Nonetheless, retired older adults suffering from these conditions who engage in light PA experience healthy aging improvements relative to those abstaining from light PA. Specifically, regardless of frequency, light PA is associated with reduced disability for retired older adults suffering neurological conditions (see the interaction effects portion of the disability domain in Table 2). Also, the influence of musculoskeletal conditions on the cognitive and physical functioning domains of healthy aging is sufficiently mitigated if retired older adults engage in daily, light PA (cognition functioning: β = *0.* 761, *p* < 0.1) or 2 to 7 days (physical functioning: β = *0.* 236 *p* < 0.1 to β = *0.* 335 *p* < 0.05). Additionally, engaging in 1—3 days per month of light PA (β = 0. 273, *p* < *0.05*) is associated with reduced disability. However, light physical activities can have deleterious effects on the physical functioning of those suffering from cardiometabolic and respiratory conditions if they engaged in one to six days (cardiometabolic: β *= -0.250*, *p* < 0.05 to β = -0.213, *p* < 0.10) and daily (respiratory: β *= -0.378*, *p* < 0.1) light physical activities per week.

**Fig. 1:**
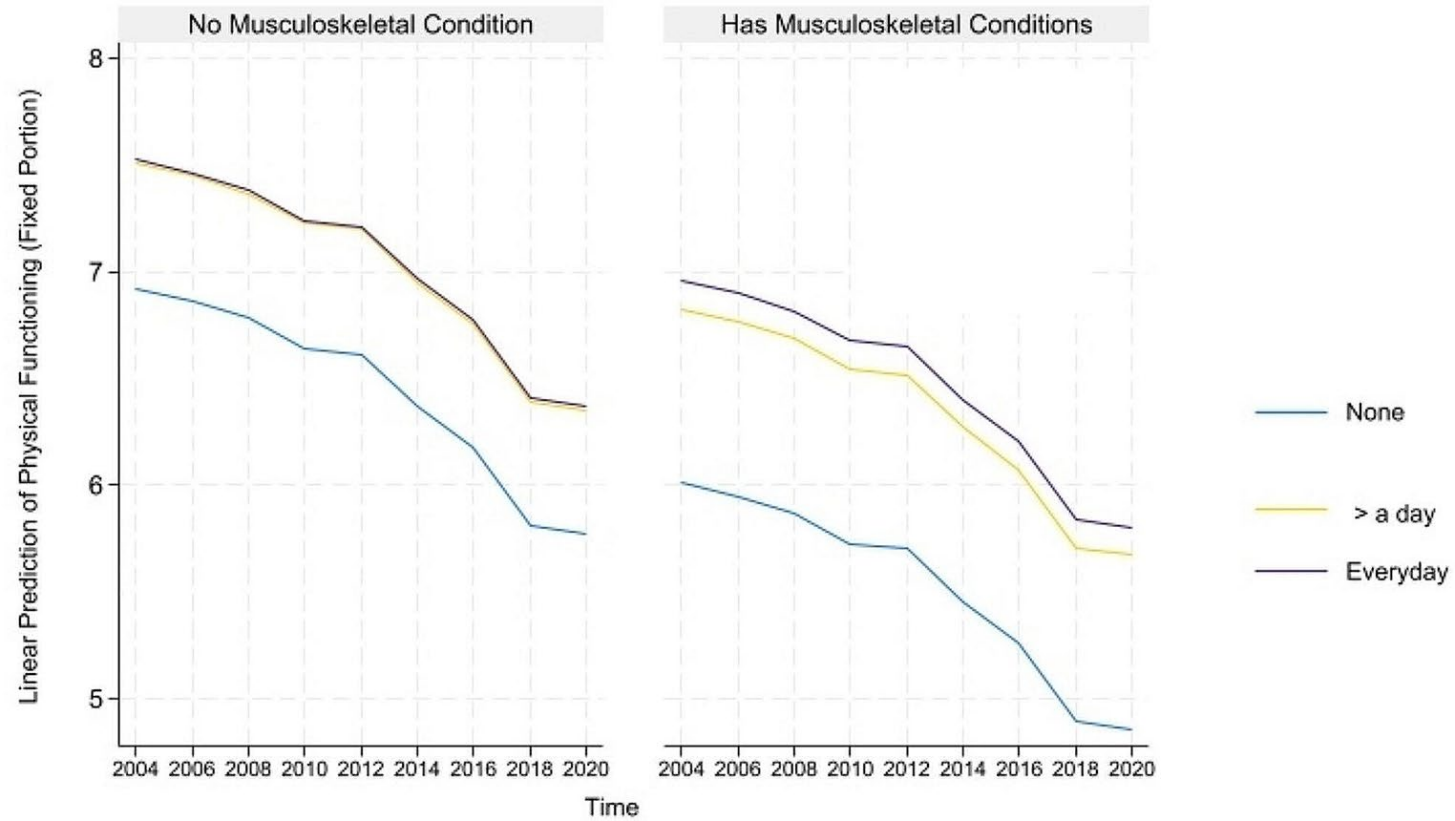
Prediction of light physical activities (and frequency) moderating the effects of musculoskeletal conditions on the predicted physical functioning domain of healthy aging

**Fig. 2:**
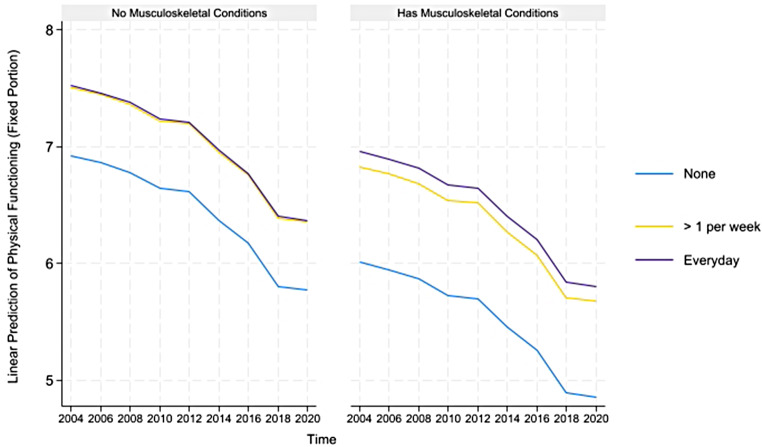
Prediction of light physical activities (and frequency) moderating the effects of neurological conditions on the predicted disability domain of healthy aging

**Fig. 3:**
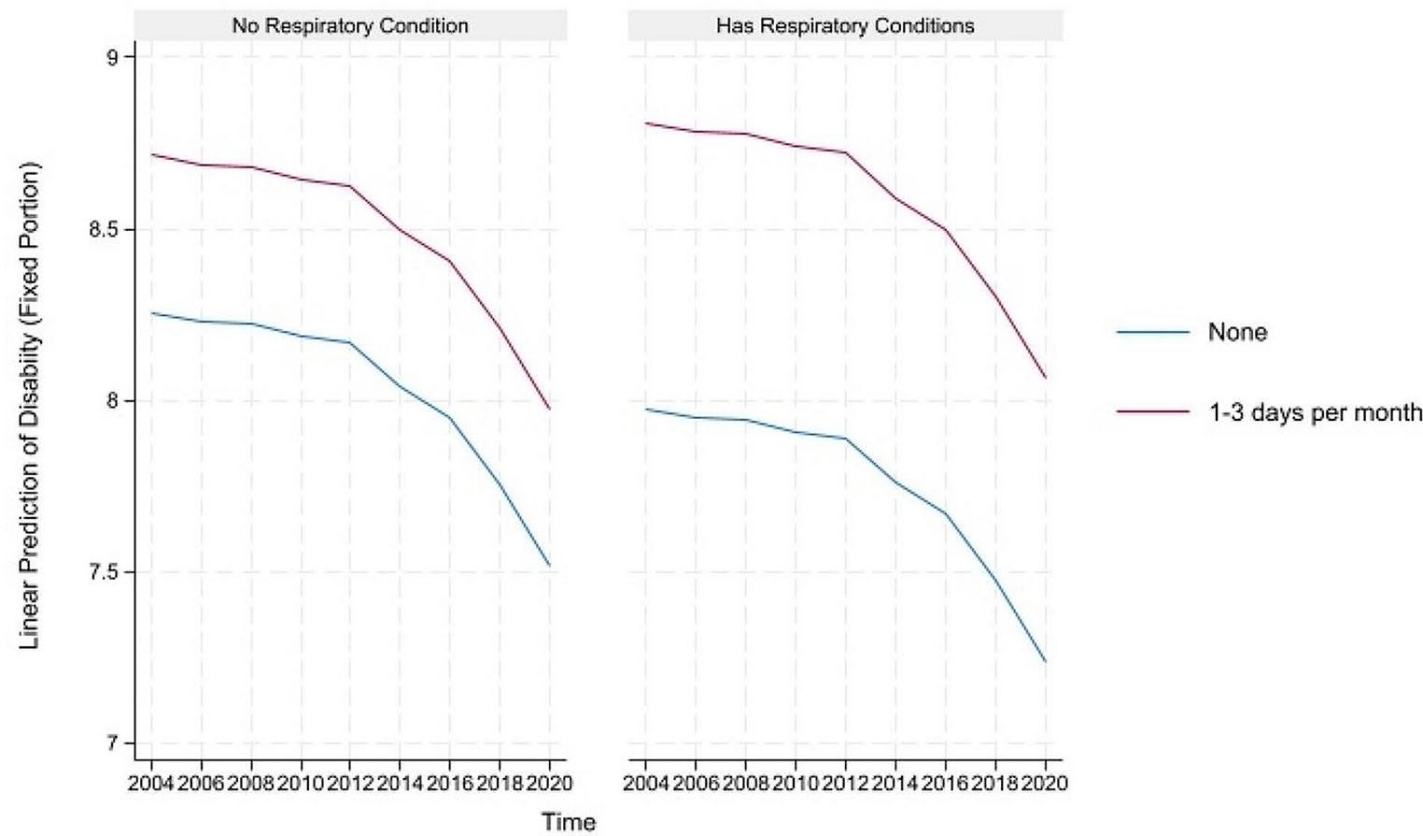
Prediction of light physical activities (and frequency) moderating the effects of respiratory conditions on the predicted disability domain of healthy aging

For concision, we depict the predicted healthy aging and counteractive effects of multimorbidity and light PA (and frequency) for portions where their direct and indirect effects are simultaneously significant in Figs. 1, 2 and 3 below. The figures on the left show that although healthy aging attainment declines with time, retired older adults engaging in light PA have better healthy aging attainment in the physical and disability domains of healthy aging. However, the plots on the right show that the differences in outcome are more apparent for the subgroup suffering from multimorbidity, where those engaging in light PA have significantly higher healthy aging attainment than those abstaining from light PA.

### The moderating effects of moderate physical activities intensities (and frequency)

Table 3 reports the mixed effects results for the moderating effects of moderate PA (and frequency) on the association between five multimorbidity classes and the physical functioning, and disability domains of healthy aging. Engaging in moderate PA intensity is significantly associated with improving physical functioning, marginally associated with better cognition. However, it does not significantly influence the disability domains of healthy aging (see the direct effects portion of moderate PA in Table 3). In contrast, retired older adults suffering cardiometabolic, neurological, musculoskeletal, and respiratory conditions experience worsening healthy aging in the domains of physical functioning (neurological: β *= -0.558*, *p* < 0.01; musculoskeletal: β *= -0.858*, *p* < 0.01; respiratory: β = -0.320, *p* < 0.01) and disability (cardiometabolic: β *= -0.124*, *p* < 0.01; neurological: β *= -0.257*, *p* < 0.01; musculoskeletal: β *= -0.285*, *p* < 0.01; respiratory: β = -0. 143, *p* < 0.05).

**Table 3:** The moderating effects of moderate physical activity intensity (and frequency) on healthy aging

	Cognitive Functioning	Physical Functioning	Disability
*Cardiometabolic Conditions*			
Yes	0.078	-0.082	-0.124***
*Physical Activity (and Frequency)*			
1—3 days per month	0.530	0.390***	0.064
1 day per week	0.615*	0.488***	0.010
> 1 day per week	0.470	0.508***	-0.054
Everyday	0.396	0.549***	-0.047
*Cardiometabolic Conditions # Physical Activity (and Frequency)*			
1—3 days per month	-0.125	-0.119	-0.045
1 day per week	0.041	-0.118	0.102*
> 1 day per week	-0.076	-0.158*	0.081
Everyday	-0.343	-0.236**	0.096
*Neurological Conditions*			
Yes	-0.193	-0.558***	-0.257***
*Neurological Conditions # Physical Activity (and Frequency)*			
1—3 days per month	-0.304	0.269**	0.067
1 day per week	-0.166	0.180	0.121*
> 1 day per week	0.259	0.251**	0.196***
Everyday	0.054	0.253*	0.227***
*Musculoskeletal Conditions*			
Yes	0.033	-0.858***	-0.285***
*Musculoskeletal Conditions # Physical Activity (and Frequency)*			
1—3 days per month	0.116	0.008	0.243***
1 day per week	0.119	0.112	0.220***
> 1 day per week	0.120	0.179*	0.241***
Everyday	0.452	0.201	0.211***
*Respiratory*			
Yes	-0.100	-0.320***	-0.143**
*Respiratory Conditions # Physical Activity (and Frequency)*			
1—3 days per month	0.253	-0.077	0.064
1 day per week	-0.047	-0.330**	-0.071
> 1 day per week	-0.218	-0.291**	-0.014
Everyday	-0.088	-0.259	-0.043
Cancer			
Yes	0.305	-0.084	-0.006
*Cancer # Physical Activity (and Frequency)*			
1—3 days per month	-0.159	-0.011	-0.039
1 day per week	-0.457	-0.083	-0.076
> 1 day per week	-0.526**	0.170*	-0.022
Everyday	-0.107	0.257*	0.011
*Race (Ref: White)*	-1.392***	-0.292***	-0.102**
*Sex (Ref: Male)*	0.949***	-0.452***	-0.010
*Education Attainment (Ref: < High School)*	0.899***	0.169***	0.016
*Age*	-0.112***	0.014*	0.007**
*Marital Status (Ref: Unmarried)*	-0.095	0.144	0.018
*Socioeconomic Status (Ref: Low SES)*	0.507***	0.092***	0.035***
*Body Mass Index*	0.026**	-0.064***	-0.017***
*Drinking (Ref: No drinking)*	0.036	0.041***	0.014**
*Smoking (Ref: No smoking)*	-0.036	-0.016	-0.105**
*Wave (Ref: 2004)*			
2006	-0.128	-0.060	-0.021
2008	0.014	-0.143**	-0.028
2010	-0.527***	-0.263***	-0.069*
2012	-0.546***	-0.311***	-0.086**
2014	-0.690***	-0.537***	-0.218***
2016	-0.946***	-0.738***	-0.309***
2018	-1.071***	-1.096***	-0.519***
2020	-1.543***	-1.143***	-0.774***
Constant	25.783***	8.158***	8.905***
Variance (Wave)	-0.059	-0.579***	-1.105***
Variance (Wave^2)	-2.367***	-2.885***	-3.159***
Variance (Constant)	0.924***	0.509***	-0.565***
Covariance (Wave, Wave^2)	-1.585***	-1.524***	-1.507***
Covariance (Wave, Constant)	-0.545***	-0.486***	-0.722***
Covariance (Wave^2, Constant)	0.416***	0.329***	0.617***
Variance (Residuals)	1.043***	0.150***	-0.384***
Number of observations	11,142	11,142	11,142

***p < 0.01, **p < 0.05, *p < 0.1

Table 3 also shows that retired older adults suffering from these conditions who engage in moderate PA have better healthy aging. For instance, retired older adults who suffer cardiometabolic conditions and engage in moderate PA once a week have reduced disability (β = 0.102, *p* < *0.1*). Also, almost all frequencies of moderate PA are associated with improving physical functioning and disability (see the interaction effects portion of the physical functioning and disability domains in Table 3) for retired older adults suffering from neurological conditions. Additionally, while the moderating influence of moderate PA on the physical functioning of retired older adults with musculoskeletal conditions is only statistically significant for retired older adults engaging in between 2 and 7 days of moderate PA per week (β = 0.179, *p* < 0.1), its moderating impact on the disability domain is statistically significant regardless of moderate PA frequency. Furthermore, the influence of cancer on the physical functioning domain is somewhat mitigated if retired older adults engage in two to daily moderate PA (β = 0.170, *p* < 0.1 to β = 0.257, *p* < *0.1*). Despite its positive influences of, too much moderate PA (one to daily moderate PA a week) can negatively impact the cognitive and physical functioning domains of healthy aging for retired older adults with cancer (cognition: β *= -0.526*, *p* < 0.05), cardiometabolic (physical functioning: β *= -0.158*, *p* < 0.1 to β = -0.236, *p* < 0.05) and respiratory conditions (physical functioning: β *= -0.330*, *p* < 0.05 to β = -0.291, *p* < 0.05).

**Fig. 4:**
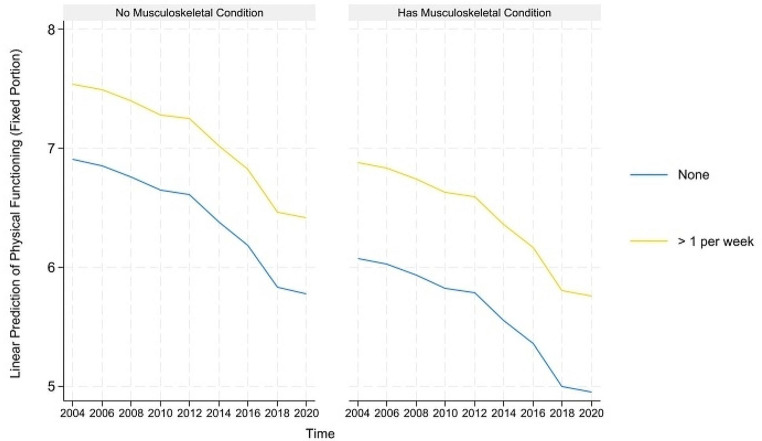
Prediction of moderate physical activities (and frequency) moderating the effects of musculoskeletal conditions on the predicted physical functioning domain of healthy aging

**Fig. 5:**
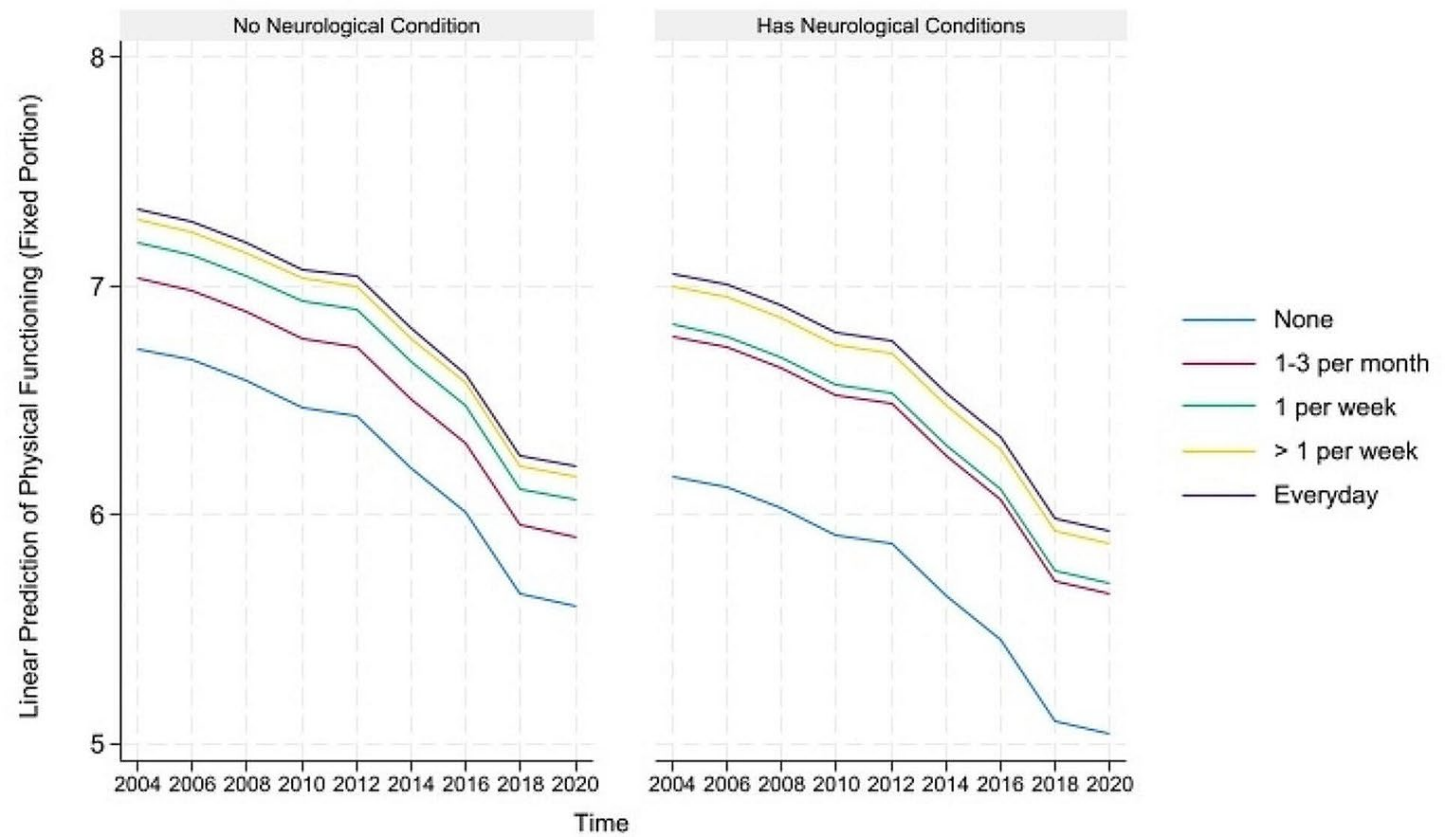
Prediction of moderate physical activities (and frequency) moderating the effects of neurological conditions on the predicted physical functioning domain of healthy aging

**Fig. 6:**
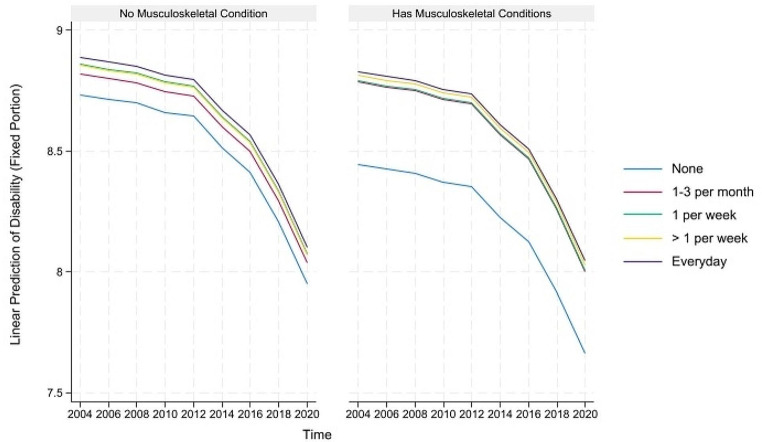
Prediction of moderate physical activities (and frequency) moderating the effects of musculoskeletal conditions on the predicted disability domain of healthy aging

**Fig. 7:**
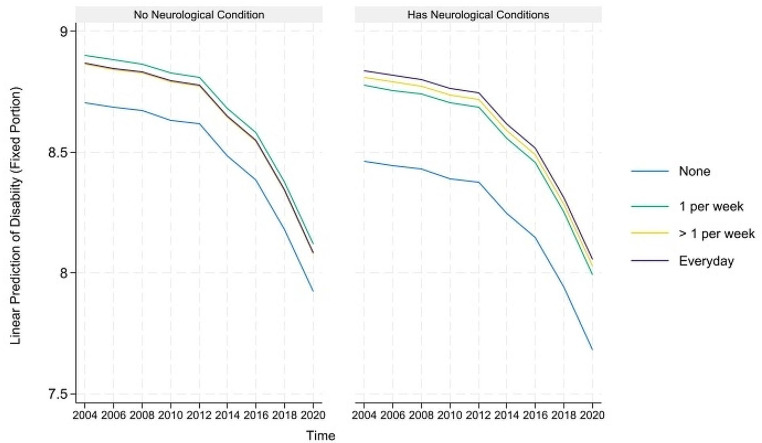
Prediction of moderate physical activities (and frequency) moderating the effects of neurological conditions on the predicted disability domain of healthy aging

**Fig. 8:**
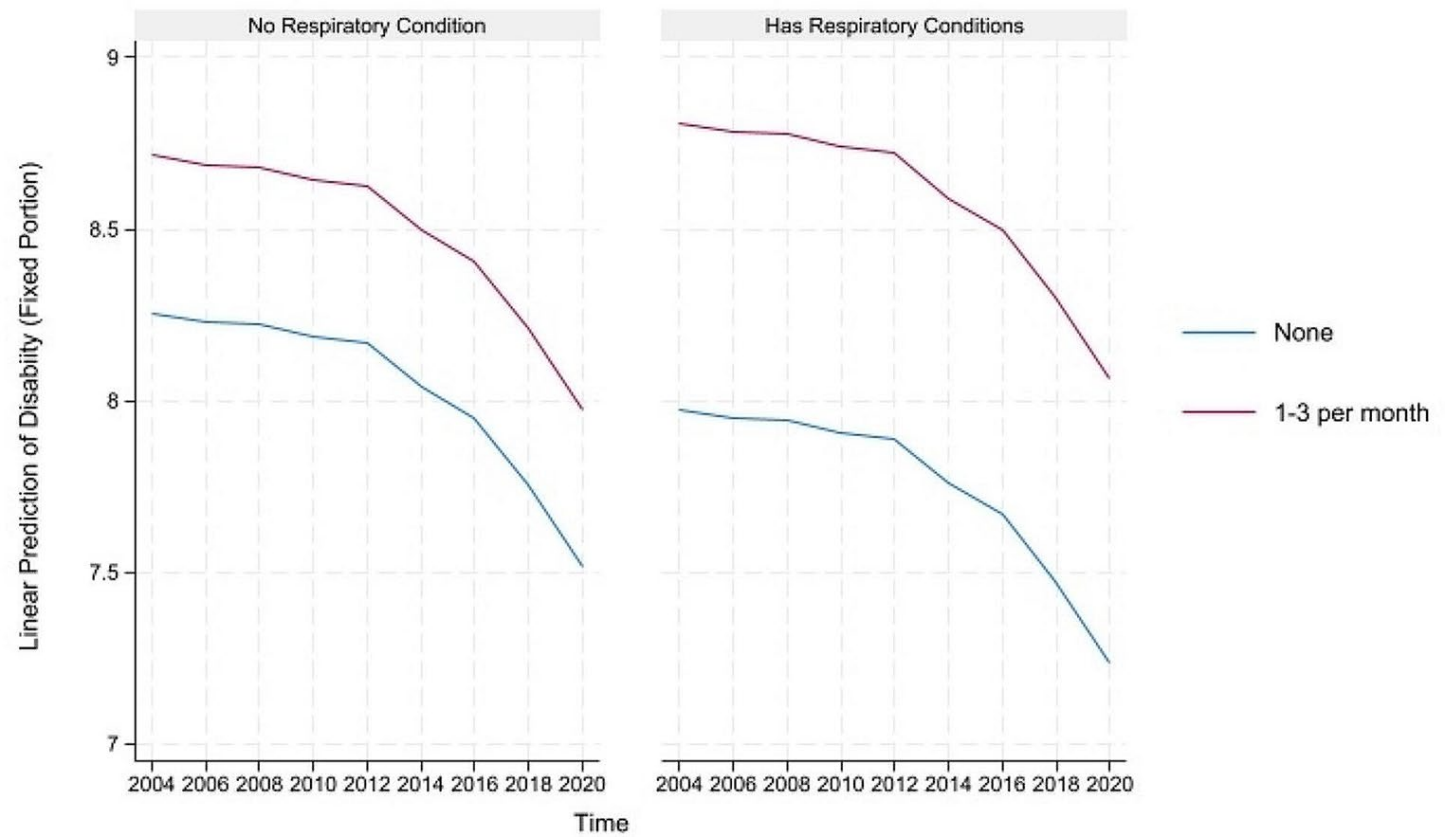
Prediction of moderate physical activities (and frequency) moderating the effects of respiratory conditions on the predicted disability domain of healthy aging

The predicted healthy aging and counteractive effects of multimorbidity and moderate PA (and frequency) for portions where their direct and indirect effects are simultaneously significant are displayed in Figs. 4, 5, 6, 7 and 8 below. Similar to the light physical activity intensities, retired older adults engaging in moderate physical activity intensities have better predicted healthy aging irrespective of their multimorbidity status, although the outcome differentials are more evident for the subgroup with multimorbidity.

### The moderating effects of vigorous physical activities intensities (and frequency)

**Fig. 9:**
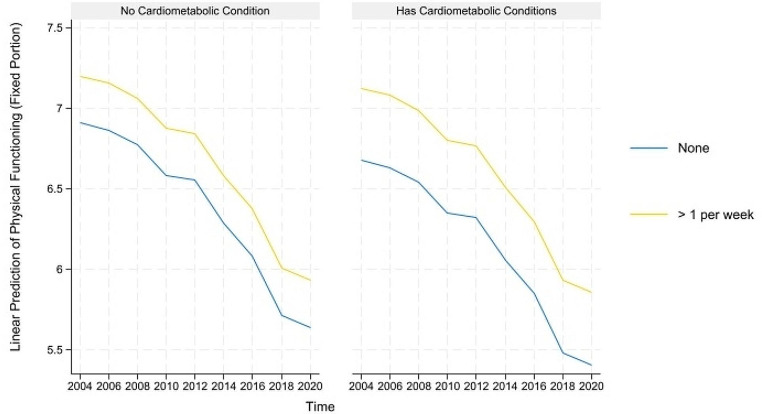
Prediction of vigorous physical activities (and frequency) moderating the effects of cardiometabolic conditions on the predicted physical functioning domain of healthy aging

The results of the impact of vigorous PA (and frequency) on multimorbidity and healthy aging are reported in Table 4. The results indicate that the influence of vigorous activities on the healthy aging of retired older adults is mainly nonsignificant, as only physical functioning is significantly influenced or improved by 2 to 6 days of vigorous physical activity (β = 0.193, *p* < *0.05*). Hence, although cardiometabolic conditions are associated with worsening physical functioning and disability (β = -0.272, *p* < 0.01 and β = -0.272, *p* < *0.01)*, vigorous physical activities are found to only improve the physical functioning of retired older adults with cardiometabolic conditions (β = *0.171*, *p* < 0.05). The pictorial representation of these effects is illustrated in Fig. 9 below. It also shows the widening outcome gap between retired older adults suffering from cardiometabolic conditions and engaging in vigorous PA intensities.

**Table 4:** The moderating effects of vigorous physical activity intensity (and frequency) on healthy aging

	Cognitive Functioning	Physical Functioning	Disability
*Cardiometabolic Conditions*			
Yes	-0.082	-0.272***	-0.091***
*Physical Activity (and Frequency)*			
1—3 days per month	0.151	0.119	0.010
1 day per week	0.148	0.112	-0.012
> 1 day per week	-0.037	0.193**	-0.057
Everyday	-0.310	0.104	-0.063
*Cardiometabolic Conditions # Physical Activity (and Frequency)*			
1—3 days per month	0.316	0.183*	0.093
1 day per week	-0.028	0.047	0.015
> 1 day per week	0.136	0.171**	0.066
Everyday	0.112	-0.016	0.005
*Neurological Conditions*			
Yes	-0.144	-0.369***	-0.152***
*Neurological Conditions # Physical Activity (and Frequency)*			
1—3 days per month	-0.443	0.083	0.125*
1 day per week	0.370	0.126	0.136**
> 1 day per week	0.030	0.031	0.100**
Everyday	0.274	0.001	0.022
*Musculoskeletal Conditions*			
Yes	0.250	-0.800***	-0.096***
*Musculoskeletal Conditions # Physical Activity (and Frequency)*			
1—3 days per month	-0.094	0.103	-0.018
1 day per week	-0.523*	0.129	0.032
> 1 day per week	0.010	0.121	0.052
Everyday	-0.332	0.041	-0.028
*Respiratory*			
Yes	-0.352	-0.455***	-0.195***
*Respiratory Conditions # Physical Activity (and Frequency)*			
1—3 days per month	0.400	0.048	0.221**
1 day per week	0.647	0.032	-0.048
> 1 day per week	0.451	-0.117	0.137
Everyday	0.357	-0.389***	-0.187**
Cancer			
Yes	-0.029	-0.007	-0.064
*Cancer # Physical Activity (and Frequency)*			
1—3 days per month	0.056	0.009	-0.005
1 day per week	0.003	0.071	0.036
> 1 day per week	-0.096	-0.008	0.084
Everyday	0.091	-0.042	0.059
*Race (Ref: White)*	-1.386***	-0.296***	-0.104**
*Sex (Ref: Male)*	0.917***	-0.456***	-0.011
*Education Attainment (Ref: < High School)*	0.910***	0.176***	0.016
*Age*	-0.111***	0.015*	0.007**
*Marital Status (Ref: Unmarried)*	-0.071	0.149	0.025
*Socioeconomic Status (Ref: Low SES)*	0.522***	0.097***	0.038***
*Body Mass Index*	0.020*	-0.069***	-0.020***
*Drinking (Ref: No drinking)*	0.034	0.040***	0.013**
*Smoking (Ref: No smoking)*	-0.055	-0.045	-0.116**
*Wave (Ref: 2004)*			
2006	-0.071	-0.049	-0.019
2008	0.041	-0.135**	-0.023
2010	-0.531***	-0.327***	-0.090**
2012	-0.558***	-0.368***	-0.105**
2014	-0.707***	-0.630***	-0.254***
2016	-0.990***	-0.839***	-0.352***
2018	-1.113***	-1.208***	-0.567***
2020	-1.535***	-1.300***	-0.779***
Constant	26.228***	8.583***	8.954***
Variance (Wave)	-0.075	-0.553***	-1.092***
Variance (Wave^2)	-2.393***	-2.852***	-3.153***
Variance (Constant)	0.934***	0.521***	-0.528***
Covariance (Wave, Wave^2)	-1.539***	-1.516***	-1.478***
Covariance (Wave, Constant)	-0.546***	-0.479***	-0.725***
Covariance (Wave^2, Constant)	0.403***	0.326***	0.599***
Variance (Residuals)	1.044***	0.154***	-0.381***
Number of observations	11,142	11,142	11,142

***p < 0.01, **p < 0.05, *p < 0.1

### The influence of the covariates, between-individual healthy aging outcomes, and sensitivity analysis

Although the magnitude of the estimated coefficientsfor the sociodemographic variables differ slightly, the signs are incredibly consistent across the nine models. For instance, although declining over time, younger white females with higher education attainment and lower BMI are estimated to have better cognition regardless of PA intensities. Notwithstanding, males with similar characteristics are estimated to have better physical functioning and ability. Furthermore, the significant variance of intercepts [variance (constant)] across all nine models indicates that the average healthy aging status differed significantly between the retired older adults population in the US.

#### Sensitivity analysis

We replicated our analysis on the survey sample with complete data (complete case analysis) for the sensitivity analysis. Tables S4 to S6 of the Additional File show that the sensitivity analysis results are quantitatively comparable to the main analysis results (with imputed data), demonstrating that our findings credibly express the healthy aging situation of the retired subpopulation of US older adults and that the imputation technique suits the data.

## Discussions

This study contributes to the ongoing quest for healthy aging enhancement by examining the moderating effects of physical activities (PA), including its intensity and frequency on the healthy aging of retired older adults living with multimorbidity in the US. Our findings provide several insights. Firstly, while there was considerable variability in baseline healthy aging between individuals, retired older adults attained favorable healthy aging in the disability and cognitive functioning domains, but fell just short of achieving health aging in the physical functioning domain. Secondly, we identified multimorbidity, particularly musculoskeletal and neurological conditions, as the most significant exacerbators of healthy aging decline, while engagement in PA was significantly associated with slowing healthy aging decline rate. Thirdly, PA moderated the impact of multimorbidity on the disability and physical functioning domains of healthy aging, though the specific effects of PA depended on its frequency, intensity, and the class of multimorbidity present.

Additionally, although neither multimorbidity nor PA significantly influenced the cognitive functioning domain in our study, engaging in ‘too much’ PA could worsen the impact of multimorbidity on healthy aging. Furthermore, we observed gender differences in healthy aging outcomes, with females demonstrating higher levels of healthy aging in cognition, while males performed better in the disability and physical functioning domains. To foster independence among older adults and alleviate functionality impairment in later life, these findings emphasizes the critical significance of formulating tailored interventions that appropriately align the intensity and frequency of physical activity to specific chronic conditions.

Based on the first hypothesis, our research confirms that older adults initially achieved healthy aging in the disability and cognitive functioning domains but marginally failed to attain adequate physical functioning at baseline. This split findings is consistent with studies demonstrating that older adults do not need to attain healthy aging across all domains [23], and suggests that while the wear and tear associated with work and daily living may not significantly impede cognition or lead to disability upon and during retirement, retired older adults may lose their ability to engage in activities requiring substantial mobility and the use of large muscles, such as prolonged sitting or heavy lifting (based on this study’s definitions of physical functioning). Moreover, retired older adults’ inability to attain physical functioning at baseline could be attributed to the prevalence of musculoskeletal conditions, mainly low-back pain, which can limit sitting and heavy lifting [51, 52]. Nevertheless, over the sixteen years in review, older adults experienced a steady decline in their capacity for healthy aging across all domains. Despite the inevitable decline in healthy aging capacity among retired older adults [5, 21], our research prove evidence that multimorbidity can exacerbate this decline, while physical activity (PA) has the potential to mitigate healthy aging deterioration over time.

Based on the second hypothesis, our analysis reveals that multimorbidity significantly hinders healthy aging in the disability and physical functioning domains, while having no substantial effects on the cognitive functioning of retired older adults. This finding aligns with existing evidence indicating that multimorbidity can have diverse impacts, the with disability and physical functioning domains being particularly susceptible to short-term pains associated with multimorbidity [12], while cognition may be more prone chronic pains resulting from multimorbidity [53]. While our findings on cognition differ from a similar study conducted on the US Health and Retirement Study (HRS) [54], we argue that their analysis may have overestimated the impact of multimorbidity because all their sample had multimorbidity, unlike our study where most individuals had no multimorbidity at baseline. Besides, the terminal year of their study is six years behind the current study, and hence, sample could differ due to new study enrollees or advances in technology (for example, smartphones and other gadgets assisting with cognitive tasks) minimizing the effect of multimorbidity on cognition [55]. Even though the association between cognition and multimorbidity is negative, its nonsignificance could be explained by research identifying age as the most significant predictor of long-term cognitive decline [56].

Additionally, musculoskeletal conditions such as back, neck, and joint pains emerge as the most significant threat to older adults’ physical functioning, while neurological conditions such as psychiatric problems pose the greatest risk to disability. These findings are supported by previous research, which has identified musculoskeletal and neurological conditions as the most prevalent and costly health problem formenting disabilities and limiting physical functioning among the US older adults and other climes [51, 52, 57–59]. Nevertheless, our study contributes further nuances by demonstrating that non-medical interventions such as physical activity play a crucial role in mitigating the influence of multimorbidity and reducing healthcare expenses. Unlike studies that operationalize multimorbidity as a simple disease count, our findings emphasizes the importance of considering the impact of specific clusters of chronic conditions on healthy aging [11].

The findings from hypotheses three and four highlight the significant role of physical activity (PA) in moderating the impact of multimorbidity on healthy aging across the examined domains. Specifically, both PA intensity and frequency played pivotal roles in moderating the impact of cardiometabolic, musculoskeletal, neurological, and respiratory conditions on physical functioning and disability domains of healthy aging. For retired older adults engaging in light PA intensity activities such as vacuuming or home repairs, participating in PA one to three days or daily per month leads to improvements in musculoskeletal, neurological, and respiratory health, as well as independence and physical functioning. Similarly, retired individuals engaging in moderate PA intensity activities such as gardening or walking also experience improvements in these health aspects across different frequencies of PA engagement. However, for those engaging in vigorous PA intensity activities such as running or cycling, improvements are primarily observed in cardiometabolic conditions and physical functioning, albeit to a lesser extent. While prior research has assessed the role of physical activity (PA) in ameliorating the impact of multimorbidity on physical and cognitive functioning in the United States and England [7, 15], our investigation extends this understanding by examining the distinct effects of varying PA intensities and frequencies on diverse chronic conditions. This nuanced approach aims to offer a more precise quantitative perspective on strategies to mitigate the influence of specific chronic conditions on healthy aging.

In summary, the significant variability in healthy aging attainment among retired older adults (significant variance of constant) underlies the need to account for these differences when addressing healthy aging concerns in the US. Accurately addressing these variations would aid in assessing the potential impact of interventions and contribute to achieving health equity. Fortunately, the dynamic nature of our findings facilitates this objective by highlighting specific frequencies and intensities of physical activity (PA) tailored to different aspects of multimorbidity and healthy aging. These indicate that PA is an essential, cost-effective, and modifiable strategy for alleviating the impact of multimorbidity on healthy aging and, hence should be encouraged.

### Limitations

This study has several limitations to consider during implementation. Firstly, although the influence is not substantial, as evidenced by the imputation summaries and sensitivity analysis, readers should consider the potential inferential bias due to data imputation. Secondly, it is essential to note that multimorbidity and physical activity intensities (and frequencies) are self-reported and may be susceptible to bias. Thirdly, we acknowledge that this study’s three healthy aging domains are not exhaustive. However, they provide a credible framework to investigate the much-needed nuances necessary for engendering the healthy aging of older adults with multimorbidity. Finally, as much as this research benefits both clinical and behavioral experts, the nature of our analysis and discussions may tilt toward the behavioral tendencies of retired older adults.

## Conclusions

This study examined the role of physical activity (PA) in enhancing the healthy aging trajectories of retired older adults with multimorbidity in the US. We find that despite significant disparities in baseline healthy aging levels, retired older adults attained healthy aging in disability and cognitive functioning but fell short of achieving favorable physical functioning. Also, while multimorbidity, especially musculoskeletal and neurological conditions, is the most significant exacerbator of healthy aging decline, PA credibly decelerates this decline. Moreover, the negative influence of multimorbidity and the moderating influence of PA depends on the PA frequency, intensity, and the nature of the chronic condition. Interestingly, our study provide evidence suggesting that excessive PA potentially exacerbates the effects of multimorbidity on healthy aging. This emphasizes the need for tailored interventions aligning PA with specific chronic conditions to preserve independence and mitigate functional decline in later life. Furthermore, gender disparities further elucidate the complexity of healthy aging outcomes, with females exhibiting superior cognitive health while males demonstrate better outcomes in disability and physical functioning. Ultimately, this sixteen-year longitudinal analysis emphasizes the potency of PA as an amenable, cost-effective tool for achieving healthy aging in the face of multimorbidity.

### Electronic supplementary material

Below is the link to the electronic supplementary material.

Supplementary Material 1

## Data Availability

Publicly available datasets were used in this study. These can be found in [Health and Retirement Study Website] at https://hrsdata.isr.umich.edu/data-products/rand-hrs-longitudinal-file-2020. Further enquiries regarding the data can be directed to the corresponding authors, who can provide them upon a reasonable request.

## Abbreviations

HRS: Health and Retirement Study
MICE: Multivariate imputed chained equation
PA: Physical Activity
