# Supplementary material for: Physical activity dynamically moderates the impact of multimorbidity on the trajectory of healthy aging over sixteen years
Source: BMC Geriatr. 2024 Jun 28;24:565. doi: 10.1186/s12877-024-05067-1 (PMC11212370; doi:10.1186/s12877-024-05067-1)
Supplement: Supplementary file 1 — Supplementary Material 1 [file 12877_2024_5067_MOESM1_ESM.docx]

Table S1. Sample Characteristics of the original data (M=0)

| Variable | Observation | | Mean | Std. dev. | Min | Max |
| --- | --- | --- | --- | --- | --- | --- |
| Race | | 11,142 | 1.169628 | .4313996 | 1 | 3 |
| Sex | | 11,142 | 1.613086 | .4870656 | 1 | 2 |
| Education Attainment | | 11,142 | 3.369144 | 1.257763 | 1 | 5 |
| Age | | 11,134 | 75.29154 | 7.910051 | 50 | 104 |
| Marital Status | | 11,133 | .7243331 | .4468698 | 0 | 1 |
| Cognition | | 9,719 | 22.44356 | 4.548732 | 1 | 35 |
| Disability | | 11,133 | 8.520075 | 1.216752 | 0 | 10 |
| Physical Functioning | | 11,134 | 6.408209 | 2.490108 | 0 | 9 |
| Cardiometabolic Condition | | 11,134 | .42231 | .4939496 | 0 | 1 |
| Neurological Condition | | 11,134 | .720855 | .4485991 | 0 | 1 |
| Musculoskeletal Condition | | 11,134 | .7007365 | .457956 | 0 | 1 |
| Respiratory Condition | | 11,134 | .1007724 | .3010406 | 0 | 1 |
| Cancer | | 11,134 | .2075624 | .4055799 | 0 | 1 |
| Light Physical Activities | | 11,123 | 3.32716 | 1.105335 | 1 | 5 |
| Moderate Physical Activities | | 11,115 | 3.081332 | 1.333544 | 1 | 5 |
| Vigorous Physical Activities | | 11,092 | 3.990534 | 1.338418 | 1 | 5 |
| Socioeconomic Status | | 11,134 | 3.431202 | 1.57266 | 0 | 5 |
| Body Mass Index | | 11,047 | 27.9087 | 5.586428 | 12.6 | 58.6 |
| Drinking Status | | 11,099 | 1.225966 | 2.172447 | 0 | 7 |
| Smoking Status | | 11,071 | .0610604 | .2394519 | 0 | 1 |

Table S2. Sample characteristics of imputation data (M=10)

| Variable | Observation | Mean | Std. dev. | Min | Max |
| --- | --- | --- | --- | --- | --- |
| Race | 11,142 | 1.169628 | .4313996 | 1 | 3 |
| Sex | 11,142 | 1.613086 | .4870656 | 1 | 2 |
| Education Attainment | 11,142 | 3.369144 | 1.257763 | 1 | 5 |
| Age | 11,142 | 75.29053 | 7.908255 | 50 | 104 |
| Marital Status | 11,142 | .7243762 | .4468481 | 0 | 1 |
| Cognition | 11,142 | 22.52043 | 4.58783 | 1 | 36.3594 |
| Disability | 11,142 | 8.520141 | 1.216816 | 0 | 10.69346 |
| Physical Functioning | 11,142 | 6.407218 | 2.491109 | 0 | 10.75433 |
| Cardiometabolic Condition | 11,142 | .4220068 | .4939018 | 0 | 1 |
| Neurological Condition | 11,142 | .7210555 | .4485003 | 0 | 1 |
| Musculoskeletal Condition | 11,142 | .7006821 | .4579798 | 0 | 1 |
| Respiratory Condition | 11,142 | .1007898 | .3010637 | 0 | 1 |
| Cancer | 11,142 | .2074134 | .4054724 | 0 | 1 |
| Light Physical Activities | 11,142 | 3.326961 | 1.105911 | 1 | 5 |
| Moderate Physical Activities | 11,142 | 3.081852 | 1.333169 | 1 | 5 |
| Vigorous Physical Activities | 11,142 | 3.990666 | 1.338601 | 1 | 5 |
| Socioeconomic Status | 11,142 | 3.431431 | 1.572618 | 0 | 5 |
| Body Mass Index | 11,142 | 27.9167 | 5.586959 | 12.6 | 58.6 |
| Drinking Status | 11,142 | 1.226171 | 2.17203 | 0 | 7 |
| Smoking Status | 11,142 | .0612996 | .2398898 | 0 | 1 |

Table S3. Sample characteristics of imputation data (M=20)

| Variable | Observation | Mean | Std. dev. | Min | Max |
| --- | --- | --- | --- | --- | --- |
| Race | 11,142 | 1.169628 | .4313996 | 1 | 3 |
| Sex | 11,142 | 1.613086 | .4870656 | 1 | 2 |
| Education Attainment | 11,142 | 3.369144 | 1.257763 | 1 | 5 |
| Age | 11,142 | 75.29053 | 7.908255 | 50 | 104 |
| Marital Status | 11,142 | .7243762 | .4468481 | 0 | 1 |
| Cognition | 11,142 | 22.52043 | 4.58783 | 1 | 36.3594 |
| Disability | 11,142 | 8.520141 | 1.216816 | 0 | 10.69346 |
| Physical Functioning | 11,142 | 6.407218 | 2.491109 | 0 | 10.75433 |
| Cardiometabolic Condition | 11,142 | .4220068 | .4939018 | 0 | 1 |
| Neurological Condition | 11,142 | .7210555 | .4485003 | 0 | 1 |
| Musculoskeletal Condition | 11,142 | .7006821 | .4579798 | 0 | 1 |
| Respiratory Condition | 11,142 | .1007898 | .3010637 | 0 | 1 |
| Cancer | 11,142 | .2074134 | .4054724 | 0 | 1 |
| Light Physical Activities | 11,142 | 3.326961 | 1.105911 | 1 | 5 |
| Moderate Physical Activities | 11,142 | 3.081852 | 1.333169 | 1 | 5 |
| Vigorous Physical Activities | 11,142 | 3.990666 | 1.338601 | 1 | 5 |
| Socioeconomic Status | 11,142 | 3.431431 | 1.572618 | 0 | 5 |
| Body Mass Index | 11,142 | 27.9167 | 5.586959 | 12.6 | 58.6 |
| Drinking Status | 11,142 | 1.226171 | 2.17203 | 0 | 7 |
| Smoking Status | 11,142 | .0608508 | .2390672 | 0 | 1 |

| Table S4. Sensitivity Results for the Moderating Effects of Light Physical Activity Intensity on Healthy Aging | | | | | | |
| --- | --- | --- | --- | --- | --- | --- |
|  | Cognitive Functioning | | Physical Functioning | | Disability | |
| *Cardiometabolic Conditions* |  |  |  |  |  |  |
| Yes | 0.220 |  | -0.051 |  | -0.078 |  |
| *Physical Activity* |  |  |  |  |  |  |
| 1—3 days per month | 0.866 | ** | 0.337 | * | 0.284 | *** |
| 1 day per week | 0.666 | ** | 0.662 | *** | 0.377 | *** |
| >1 day per week | 0.468 |  | 0.617 | *** | 0.318 | *** |
| Everyday | 0.678 | * | 0.586 | *** | 0.280 | *** |
| *Cardiometabolic Conditions # Physical Activity* |  |  |  |  |  |  |
| 1—3 days per month | -0.296 |  | 0.007 |  | 0.032 |  |
| 1 day per week | -0.027 |  | -0.208 | * | -0.021 |  |
| >1 day per week | -0.319 |  | -0.178 |  | 0.021 |  |
| Everyday | -0.391 |  | -0.077 |  | 0.086 |  |
| *Neurological Conditions* |  |  |  |  |  |  |
| Yes | -0.156 |  | -0.329 | ** | -0.315 | *** |
| *Neurological Conditions # Physical Activity* |  |  |  |  |  |  |
| 1—3 days per month | -0.070 |  | 0.002 |  | 0.217 | ** |
| 1 day per week | -0.045 |  | -0.089 |  | 0.204 | ** |
| >1 day per week | 0.237 |  | 0.057 |  | 0.267 | *** |
| Everyday | -0.150 |  | 0.074 |  | 0.303 | *** |
| *Musculoskeletal Conditions* |  |  |  |  |  |  |
| Yes | -0.129 |  | -0.914 | *** | -0.163 | ** |
| *Musculoskeletal Conditions # Physical Activity* |  |  |  |  |  |  |
| 1—3 days per month | -0.209 |  | 0.119 |  | -0.004 |  |
| 1 day per week | -0.053 |  | 0.138 |  | 0.074 |  |
| >1 day per week | 0.412 |  | 0.234 | * | 0.106 |  |
| Everyday | 0.746 | ** | 0.348 | ** | 0.124 |  |
| *Respiratory* |  |  |  |  |  |  |
| Yes | -0.430 |  | -0.430 | *** | -0.280 | *** |
| *Respiratory Conditions # Physical Activity* |  |  |  |  |  |  |
| 1—3 days per month | 0.282 |  | -0.136 |  | 0.373 | *** |
| 1 day per week | 0.438 |  | 0.053 |  | 0.141 |  |
| >1 day per week | 0.265 |  | -0.147 |  | 0.081 |  |
| Everyday | -0.039 |  | -0.365 | * | 0.069 |  |
| Cancer |  |  |  |  |  |  |
| Yes | -0.294 |  | -0.040 |  | 0.006 |  |
| *Cancer # Physical Activity* |  |  |  |  |  |  |
| 1—3 days per month | 0.500 |  | 0.103 |  | 0.040 |  |
| 1 day per week | 0.487 | * | -0.034 |  | -0.067 |  |
| >1 day per week | 0.305 |  | 0.066 |  | -0.031 |  |
| Everyday | 0.210 |  | 0.142 |  | -0.050 |  |
| *Race (Ref: White)* | -1.676 | *** | -0.291 | *** | -0.097 | ** |
| *Sex (Ref: Male)* | 1.013 | *** | -0.561 | *** | -0.065 | * |
| *Education Attainment (Ref: < High School)* | 1.074 | *** | 0.179 | *** | 0.015 |  |
| *Age* | -0.116 | *** | 0.012 |  | 0.006 | ** |
| *Marital Status (Ref: Unmarried)* | 0.027 |  | 0.135 |  | 0.018 |  |
| *Socioeconomic Status (Ref: Low SES)* | 0.362 | *** | 0.101 | *** | 0.037 | *** |
| *Body Mass Index* | 0.025 | ** | -0.073 | *** | -0.020 | *** |
| *Drinking (Ref: No drinking)* | 0.021 |  | 0.038 | *** | 0.010 | * |
| *Smoking (Ref: No smoking)* | 0.023 |  | -0.039 |  | -0.118 | ** |
| *Wave (Ref: 2004)* |  |  |  |  |  |  |
| 2006 | -0.158 |  | -0.061 |  | -0.026 |  |
| 2008 | 0.033 |  | -0.141 | ** | -0.032 |  |
| 2010 | -0.585 | *** | -0.283 | *** | -0.068 | * |
| 2012 | -0.609 | *** | -0.310 | *** | -0.086 | ** |
| 2014 | -0.744 | *** | -0.553 | *** | -0.215 | *** |
| 2016 | -1.006 | *** | -0.751 | *** | -0.308 | *** |
| 2018 | -1.033 | *** | -1.118 | *** | -0.504 | *** |
| 2020 | -1.567 | *** | -1.152 | *** | -0.741 | *** |
| Constant | 26.089 | *** | 8.522 | *** | 8.729 | *** |
| Variance (Wave) | 0.276 |  | 0.320 |  | 0.113 |  |
| Variance (Wave^2) | 0.005 |  | 0.003 |  | 0.002 |  |
| Variance (Constant) | 5.920 |  | 2.794 |  | 0.299 |  |
| Covariance (Wave, Wave^2) | -0.030 | ** | -0.029 | *** | -0.013 | *** |
| Covariance (Wave, Constant) | -0.276 |  | -0.417 | *** | -0.115 | *** |
| Covariance (Wave^2, Constant) | 0.030 |  | 0.029 | *** | 0.013 | *** |
| Variance (Residuals) | 6.384 |  | 1.355 |  | 0.456 |  |
| Number of observations | 9554 |  | 10930 |  | 10929 |  |
| *** p<.01, ** p<.05, * p<.1 | | | | | | |

| Table S5. Sensitivity Results for the Moderating Effects of Moderate Physical Activity Intensity on Healthy Aging | | | | | | |
| --- | --- | --- | --- | --- | --- | --- |
|  | Cognitive Functioning | | Physical Function | | Disability | |
| *Cardiometabolic Conditions* |  |  |  |  |  |  |
| Yes | 0.099 |  | -0.040 |  | -0.119 | ** |
| *Physical Activity* |  |  |  |  |  |  |
| 1—3 days per month | 0.548 | * | 0.373 | *** | 0.052 |  |
| 1 day per week | 0.709 | ** | 0.507 | *** | 0.023 |  |
| >1 day per week | 0.502 | * | 0.512 | *** | -0.044 |  |
| Everyday | 0.453 |  | 0.550 | *** | -0.036 |  |
| *Cardiometabolic Conditions # Physical Activity* |  |  |  |  |  |  |
| 1—3 days per month | -0.087 |  | -0.124 |  | -0.049 |  |
| 1 day per week | 0.001 |  | -0.136 |  | 0.098 | * |
| >1 day per week | -0.111 |  | -0.180 | ** | 0.079 |  |
| Everyday | -0.413 |  | -0.264 | ** | 0.093 |  |
| *Neurological Conditions* |  |  |  |  |  |  |
| Yes | -0.117 |  | -0.556 | *** | -0.242 | *** |
| *Neurological Conditions # Physical Activity* |  |  |  |  |  |  |
| 1—3 days per month | -0.467 |  | 0.307 | ** | 0.077 |  |
| 1 day per week | -0.180 |  | 0.196 | * | 0.116 | * |
| >1 day per week | 0.200 |  | 0.267 | *** | 0.188 | *** |
| Everyday | 0.030 |  | 0.279 | ** | 0.212 | *** |
| *Musculoskeletal Conditions* |  |  |  |  |  |  |
| Yes | -0.003 |  | -0.827 | *** | -0.288 | *** |
| *Musculoskeletal Conditions # Physical Activity* |  |  |  |  |  |  |
| 1—3 days per month | 0.194 |  | -0.001 |  | 0.255 | *** |
| 1 day per week | 0.039 |  | 0.091 |  | 0.219 | *** |
| >1 day per week | 0.131 |  | 0.170 | * | 0.248 | *** |
| Everyday | 0.420 |  | 0.196 |  | 0.230 | *** |
| *Respiratory* |  |  |  |  |  |  |
| Yes | -0.031 |  | -0.341 | *** | -0.156 | ** |
| *Respiratory Conditions # Physical Activity* |  |  |  |  |  |  |
| 1—3 days per month | 0.111 |  | -0.065 |  | 0.068 |  |
| 1 day per week | -0.149 |  | -0.315 | ** | -0.062 |  |
| >1 day per week | -0.383 |  | -0.245 | * | 0.013 |  |
| Everyday | -0.004 |  | -0.239 |  | -0.029 |  |
| Cancer |  |  |  |  |  |  |
| Yes | 0.458 | ** | -0.072 |  | -0.000 |  |
| *Cancer # Physical Activity* |  |  |  |  |  |  |
| 1—3 days per month | -0.290 |  | -0.050 |  | -0.037 |  |
| 1 day per week | -0.592 | ** | -0.091 |  | -0.073 |  |
| >1 day per week | -0.656 | *** | 0.161 |  | -0.030 |  |
| Everyday | -0.261 |  | 0.268 | * | 0.006 |  |
| *Race (Ref: White)* | -1.663 | *** | -0.300 | *** | -0.098 | ** |
| *Sex (Ref: Male)* | 1.120 | *** | -0.450 | *** | -0.014 |  |
| *Education Attainment (Ref: < High School)* | 1.072 | *** | 0.171 | *** | 0.018 |  |
| *Age* | -0.115 | *** | 0.012 |  | 0.007 | ** |
| *Marital Status (Ref: Unmarried)* | 0.048 |  | 0.141 |  | 0.017 |  |
| *Socioeconomic Status (Ref: Low SES)* | 0.363 | *** | 0.097 | *** | 0.034 | *** |
| *Body Mass Index* | 0.028 | ** | -0.068 | *** | -0.018 | *** |
| *Drinking (Ref: No drinking)* | 0.027 |  | 0.040 | *** | 0.013 | ** |
| *Smoking (Ref: No smoking)* | 0.056 |  | 0.019 |  | -0.098 | ** |
| *Wave (Ref: 2004)* |  |  |  |  |  |  |
| 2006 | -0.150 |  | -0.052 |  | -0.020 |  |
| 2008 | 0.015 |  | -0.144 | ** | -0.035 |  |
| 2010 | -0.581 | *** | -0.259 | *** | -0.073 | ** |
| 2012 | -0.603 | *** | -0.294 | *** | -0.090 | ** |
| 2014 | -0.751 | *** | -0.522 | *** | -0.219 | *** |
| 2016 | -1.022 | *** | -0.716 | *** | -0.319 | *** |
| 2018 | -1.076 | *** | -1.077 | *** | -0.525 | *** |
| 2020 | -1.649 | *** | -1.125 | *** | -0.783 | *** |
| Constant | 25.895 | *** | 8.390 | *** | 8.921 | *** |
| Variance (Wave) | 0.292 |  | 0.308 |  | 0.120 |  |
| Variance (Wave^2) | 0.005 |  | 0.003 |  | 0.002 |  |
| Variance (Constant) | 5.869 |  | 2.752 |  | 0.320 |  |
| Covariance (Wave, Wave^2) | -0.032 | ** | -0.028 | *** | -0.014 | *** |
| Covariance (Wave, Constant) | -0.270 |  | -0.413 | *** | -0.122 | *** |
| Covariance (Wave^2, Constant) | 0.031 |  | 0.028 | *** | 0.014 | *** |
| Variance (Residuals) | 6.380 |  | 1.344 |  | 0.455 |  |
| Number of observations | 9542 |  | 10921 |  | 10920 |  |
| *** p<.01, ** p<.05, * p<.1 | | | | | | |

| Table S6. Sensitivity Results for the Moderating Effects of Vigorous Physical Activity Intensity on Healthy Aging | | | | | | |
| --- | --- | --- | --- | --- | --- | --- |
|  | Cognitive Functioning | | Physical Functioning | | Disability | |
| *Cardiometabolic Conditions* |  |  |  |  |  |  |
| Yes | -0.056 |  | -0.233 | *** | -0.085 | ** |
| *Physical Activity* |  |  |  |  |  |  |
| 1—3 days per month | 0.332 |  | 0.144 |  | 0.024 |  |
| 1 day per week | 0.169 |  | 0.132 |  | 0.002 |  |
| >1 day per week | -0.037 |  | 0.196 | ** | -0.050 |  |
| Everyday | -0.275 |  | 0.082 |  | -0.057 |  |
| *Cardiometabolic Conditions # Physical Activity* |  |  |  |  |  |  |
| 1—3 days per month | 0.296 |  | 0.140 |  | 0.084 |  |
| 1 day per week | -0.080 |  | 0.036 |  | 0.011 |  |
| >1 day per week | 0.162 |  | 0.158 | * | 0.064 |  |
| Everyday | 0.076 |  | -0.037 |  | -0.034 |  |
| *Neurological Conditions* |  |  |  |  |  |  |
| Yes | -0.114 |  | -0.352 | *** | -0.139 | *** |
| *Neurological Conditions # Physical Activity* |  |  |  |  |  |  |
| 1—3 days per month | -0.599 | ** | 0.084 |  | 0.115 | * |
| 1 day per week | 0.421 | * | 0.111 |  | 0.126 | ** |
| >1 day per week | 0.028 |  | 0.044 |  | 0.097 | ** |
| Everyday | 0.330 |  | 0.012 |  | 0.014 |  |
| *Musculoskeletal Conditions* |  |  |  |  |  |  |
| Yes | 0.203 |  | -0.775 | *** | -0.090 | ** |
| *Musculoskeletal Conditions # Physical Activity* |  |  |  |  |  |  |
| 1—3 days per month | -0.098 |  | 0.090 |  | -0.027 |  |
| 1 day per week | -0.463 | * | 0.114 |  | 0.026 |  |
| >1 day per week | -0.022 |  | 0.105 |  | 0.051 |  |
| Everyday | -0.483 | * | 0.046 |  | -0.013 |  |
| *Respiratory* |  |  |  |  |  |  |
| Yes | -0.321 |  | -0.465 | *** | -0.196 | *** |
| *Respiratory Conditions # Physical Activity* |  |  |  |  |  |  |
| 1—3 days per month | 0.306 |  | 0.072 |  | 0.224 | ** |
| 1 day per week | 0.793 | * | 0.041 |  | -0.053 |  |
| >1 day per week | 0.478 |  | -0.082 |  | 0.136 |  |
| Everyday | 0.443 |  | -0.378 | *** | -0.201 | ** |
| Cancer |  |  |  |  |  |  |
| Yes | 0.058 |  | -0.004 |  | -0.063 |  |
| *Cancer # Physical Activity* |  |  |  |  |  |  |
| 1—3 days per month | -0.074 |  | -0.002 |  | -0.008 |  |
| 1 day per week | -0.079 |  | 0.086 |  | 0.039 |  |
| >1 day per week | -0.113 |  | -0.006 |  | 0.082 |  |
| Everyday | -0.066 |  | -0.050 |  | 0.039 |  |
| *Race (Ref: White)* | -1.685 | *** | -0.300 | *** | -0.096 | ** |
| *Sex (Ref: Male)* | 1.095 | *** | -0.456 | *** | -0.016 |  |
| *Education Attainment (Ref: < High School)* | 1.077 | *** | 0.177 | *** | 0.018 |  |
| *Age* | -0.116 | *** | 0.013 | * | 0.007 | ** |
| *Marital Status (Ref: Unmarried)* | 0.040 |  | 0.149 |  | 0.024 |  |
| *Socioeconomic Status (Ref: Low SES)* | 0.382 | *** | 0.103 | *** | 0.037 | *** |
| *Body Mass Index* | 0.020 | * | -0.074 | *** | -0.022 | *** |
| *Drinking (Ref: No drinking)* | 0.026 |  | 0.039 | *** | 0.012 | ** |
| *Smoking (Ref: No smoking)* | 0.011 |  | -0.006 |  | -0.110 | ** |
| *Wave (Ref: 2004)* |  |  |  |  |  |  |
| 2006 | -0.135 |  | -0.046 |  | -0.019 |  |
| 2008 | 0.054 |  | -0.137 | ** | -0.029 |  |
| 2010 | -0.588 | *** | -0.327 | *** | -0.093 | ** |
| 2012 | -0.611 | *** | -0.355 | *** | -0.109 | *** |
| 2014 | -0.773 | *** | -0.621 | *** | -0.260 | *** |
| 2016 | -1.063 | *** | -0.827 | *** | -0.363 | *** |
| 2018 | -1.113 | *** | -1.195 | *** | -0.573 | *** |
| 2020 | -1.503 | *** | -1.268 | *** | -0.774 | *** |
| Constant | 26.603 | *** | 8.808 | *** | 8.988 | *** |
| Variance (Wave) | 0.272 |  | 0.326 |  | 10899 |  |
| Variance (Wave^2) | 0.005 |  | 0.003 |  | 0.002 |  |
| Variance (Constant) | 5.859 |  | 2.821 |  | 0.337 |  |
| Covariance (Wave, Wave^2) | -0.029 | ** | -0.030 | *** | -0.014 | *** |
| Covariance (Wave, Constant) | -0.258 |  | -0.425 | *** | -0.125 | *** |
| Covariance (Wave^2, Constant) | 0.028 |  | 0.030 | *** | 0.014 | *** |
| Variance (Residuals) | 6.386 |  | 1.360 |  | 0.459 |  |
| Number of observations | 9523 |  | 10900 |  | 10899 |  |
| *** p<.01, ** p<.05, * p<.1 | | | | | | |

Table S7. Complete Sample Characteristics, by Wave.

|  | Wave | | | | | | | | | |
| --- | --- | --- | --- | --- | --- | --- | --- | --- | --- | --- |
|  | 2004 | 2006 | 2008 | 2010 | 2012 | 2014 | 2016 | 2018 | 2020 | Total |
| N | 1,238 (11.1%) | 1,238 (11.1%) | 1,238 (11.1%) | 1,238 (11.1%) | 1,238 (11.1%) | 1,238 (11.1%) | 1,238 (11.1%) | 1,238 (11.1%) | 1,238 (11.1%) | 11,142 (100.0%) |
| ***Healthy Aging Domains*** |  |  |  |  |  |  |  |  |  |  |
| *Cognition Functioning* | 23.865 (4.010) | 23.626 (4.121) | 23.652 (4.016) | 22.747 (4.122) | 22.556 (4.228) | 22.224 (4.494) | 21.740 (4.660) | 21.310 (4.937) | 20.628 (5.098) | 22.444 (4.549) |
| *Disability* |  |  |  |  |  |  |  |  |  |  |
| 0 | 0 (0.0%) | 0 (0.0%) | 0 (0.0%) | 0 (0.0%) | 0 (0.0%) | 2 (0.2%) | 2 (0.2%) | 4 (0.3%) | 9 (0.7%) | 17 (0.2%) |
| 1 | 0 (0.0%) | 1 (0.1%) | 0 (0.0%) | 1 (0.1%) | 0 (0.0%) | 2 (0.2%) | 2 (0.2%) | 7 (0.6%) | 9 (0.7%) | 22 (0.2%) |
| 2 | 0 (0.0%) | 0 (0.0%) | 1 (0.1%) | 4 (0.3%) | 4 (0.3%) | 3 (0.2%) | 5 (0.4%) | 7 (0.6%) | 15 (1.2%) | 39 (0.4%) |
| 3 | 6 (0.5%) | 1 (0.1%) | 3 (0.2%) | 1 (0.1%) | 6 (0.5%) | 8 (0.6%) | 7 (0.6%) | 15 (1.2%) | 32 (2.6%) | 79 (0.7%) |
| 4 | 7 (0.6%) | 5 (0.4%) | 4 (0.3%) | 6 (0.5%) | 7 (0.6%) | 6 (0.5%) | 19 (1.5%) | 24 (1.9%) | 26 (2.1%) | 104 (0.9%) |
| 5 | 8 (0.6%) | 11 (0.9%) | 13 (1.1%) | 18 (1.5%) | 11 (0.9%) | 25 (2.0%) | 31 (2.5%) | 48 (3.9%) | 53 (4.3%) | 218 (2.0%) |
| 6 | 16 (1.3%) | 17 (1.4%) | 17 (1.4%) | 16 (1.3%) | 24 (1.9%) | 37 (3.0%) | 38 (3.1%) | 52 (4.2%) | 51 (4.1%) | 268 (2.4%) |
| 7 | 28 (2.3%) | 39 (3.2%) | 33 (2.7%) | 47 (3.8%) | 39 (3.2%) | 55 (4.4%) | 55 (4.4%) | 72 (5.8%) | 97 (7.8%) | 465 (4.2%) |
| 8 | 74 (6.0%) | 103 (8.3%) | 104 (8.5%) | 111 (9.0%) | 123 (9.9%) | 141 (11.4%) | 164 (13.2%) | 146 (11.8%) | 177 (14.3%) | 1,143 (10.3%) |
| 9 | 1,099 (88.8%) | 1,060 (85.7%) | 1,055 (85.8%) | 1,034 (83.5%) | 1,024 (82.7%) | 959 (77.5%) | 914 (73.8%) | 863 (69.7%) | 768 (62.0%) | 8,776 (78.8%) |
| 10 | 0 (0.0%) | 0 (0.0%) | 0 (0.0%) | 0 (0.0%) | 0 (0.0%) | 0 (0.0%) | 1 (0.1%) | 0 (0.0%) | 1 (0.1%) | 2 (0.0%) |
| *Physical Functioning* |  |  |  |  |  |  |  |  |  |  |
| 0 | 7 (0.6%) | 5 (0.4%) | 9 (0.7%) | 11 (0.9%) | 11 (0.9%) | 23 (1.9%) | 29 (2.3%) | 28 (2.3%) | 38 (3.1%) | 161 (1.4%) |
| 1 | 19 (1.5%) | 26 (2.1%) | 33 (2.7%) | 32 (2.6%) | 35 (2.8%) | 40 (3.2%) | 49 (4.0%) | 89 (7.2%) | 89 (7.2%) | 412 (3.7%) |
| 2 | 28 (2.3%) | 45 (3.6%) | 33 (2.7%) | 56 (4.5%) | 50 (4.0%) | 62 (5.0%) | 62 (5.0%) | 87 (7.0%) | 95 (7.7%) | 518 (4.7%) |
| 3 | 61 (4.9%) | 50 (4.0%) | 62 (5.0%) | 62 (5.0%) | 69 (5.6%) | 97 (7.8%) | 109 (8.8%) | 103 (8.3%) | 96 (7.8%) | 709 (6.4%) |
| 4 | 75 (6.1%) | 64 (5.2%) | 63 (5.1%) | 81 (6.5%) | 89 (7.2%) | 85 (6.9%) | 83 (6.7%) | 106 (8.6%) | 119 (9.6%) | 765 (6.9%) |
| 5 | 79 (6.4%) | 104 (8.4%) | 97 (7.9%) | 110 (8.9%) | 108 (8.7%) | 114 (9.2%) | 129 (10.4%) | 111 (9.0%) | 122 (9.9%) | 974 (8.7%) |
| 6 | 113 (9.1%) | 125 (10.1%) | 158 (12.8%) | 124 (10.0%) | 158 (12.8%) | 126 (10.2%) | 148 (12.0%) | 143 (11.6%) | 119 (9.6%) | 1,214 (10.9%) |
| 7 | 172 (13.9%) | 163 (13.2%) | 156 (12.7%) | 182 (14.7%) | 157 (12.7%) | 180 (14.5%) | 171 (13.8%) | 170 (13.7%) | 151 (12.2%) | 1,502 (13.5%) |
| 8 | 248 (20.0%) | 230 (18.6%) | 223 (18.1%) | 235 (19.0%) | 197 (15.9%) | 188 (15.2%) | 185 (14.9%) | 188 (15.2%) | 171 (13.8%) | 1,865 (16.8%) |
| 9 | 436 (35.2%) | 426 (34.4%) | 396 (32.2%) | 345 (27.9%) | 364 (29.4%) | 323 (26.1%) | 273 (22.1%) | 213 (17.2%) | 238 (19.2%) | 3,014 (27.1%) |
| ***Multimorbidity*** |  |  |  |  |  |  |  |  |  |  |
| Cardiometabolic Condition |  |  |  |  |  |  |  |  |  |  |
| No | 924 (74.6%) | 878 (70.9%) | 831 (67.6%) | 768 (62.0%) | 717 (57.9%) | 661 (53.4%) | 601 (48.5%) | 547 (44.2%) | 505 (40.8%) | 6,432 (57.8%) |
| Yes | 314 (25.4%) | 360 (29.1%) | 399 (32.4%) | 470 (38.0%) | 521 (42.1%) | 577 (46.6%) | 637 (51.5%) | 691 (55.8%) | 733 (59.2%) | 4,702 (42.2%) |
| Neurological Conditions |  |  |  |  |  |  |  |  |  |  |
| No | 532 (43.0%) | 469 (37.9%) | 414 (33.7%) | 360 (29.1%) | 328 (26.5%) | 298 (24.1%) | 261 (21.1%) | 237 (19.1%) | 209 (16.9%) | 3,108 (27.9%) |
| Yes | 706 (57.0%) | 769 (62.1%) | 816 (66.3%) | 878 (70.9%) | 910 (73.5%) | 940 (75.9%) | 977 (78.9%) | 1,001 (80.9%) | 1,029 (83.1%) | 8,026 (72.1%) |
| Musculoskeletal Condition |  |  |  |  |  |  |  |  |  |  |
| No | 518 (41.8%) | 474 (38.3%) | 427 (34.7%) | 399 (32.2%) | 375 (30.3%) | 339 (27.4%) | 296 (23.9%) | 265 (21.4%) | 239 (19.3%) | 3,332 (29.9%) |
| Yes | 720 (58.2%) | 764 (61.7%) | 803 (65.3%) | 839 (67.8%) | 863 (69.7%) | 899 (72.6%) | 942 (76.1%) | 973 (78.6%) | 999 (80.7%) | 7,802 (70.1%) |
| Respiratory Conditions |  |  |  |  |  |  |  |  |  |  |
| No | 1,169 (94.4%) | 1,160 (93.7%) | 1,138 (92.5%) | 1,132 (91.4%) | 1,120 (90.5%) | 1,101 (88.9%) | 1,085 (87.6%) | 1,062 (85.8%) | 1,045 (84.4%) | 10,012 (89.9%) |
| Yes | 69 (5.6%) | 78 (6.3%) | 92 (7.5%) | 106 (8.6%) | 118 (9.5%) | 137 (11.1%) | 153 (12.4%) | 176 (14.2%) | 193 (15.6%) | 1,122 (10.1%) |
| Cancer |  |  |  |  |  |  |  |  |  |  |
| No | 1,074 (86.8%) | 1,058 (85.5%) | 1,032 (83.9%) | 1,003 (81.0%) | 983 (79.4%) | 956 (77.2%) | 930 (75.1%) | 908 (73.3%) | 879 (71.0%) | 8,823 (79.2%) |
| Yes | 164 (13.2%) | 180 (14.5%) | 198 (16.1%) | 235 (19.0%) | 255 (20.6%) | 282 (22.8%) | 308 (24.9%) | 330 (26.7%) | 359 (29.0%) | 2,311 (20.8%) |
| ***Light Physical Activity*** |  |  |  |  |  |  |  |  |  |  |
| None | 53 (4.3%) | 55 (4.4%) | 67 (5.4%) | 109 (8.8%) | 104 (8.4%) | 140 (11.3%) | 181 (14.6%) | 225 (18.2%) | 306 (24.8%) | 1,240 (11.1%) |
| 1—3 days per month | 65 (5.3%) | 65 (5.3%) | 65 (5.3%) | 103 (8.3%) | 110 (8.9%) | 112 (9.1%) | 111 (9.0%) | 108 (8.7%) | 128 (10.4%) | 867 (7.8%) |
| 1 day per week | 283 (22.9%) | 271 (21.9%) | 296 (24.1%) | 323 (26.1%) | 373 (30.2%) | 381 (30.8%) | 397 (32.1%) | 350 (28.3%) | 339 (27.5%) | 3,013 (27.1%) |
| > 1 day per week | 743 (60.0%) | 685 (55.3%) | 642 (52.2%) | 611 (49.4%) | 565 (45.7%) | 528 (42.7%) | 469 (37.9%) | 433 (35.1%) | 344 (27.9%) | 5,020 (45.1%) |
| Everyday | 94 (7.6%) | 162 (13.1%) | 160 (13.0%) | 91 (7.4%) | 85 (6.9%) | 76 (6.1%) | 80 (6.5%) | 119 (9.6%) | 116 (9.4%) | 983 (8.8%) |
| ***Moderate Physical Activity*** |  |  |  |  |  |  |  |  |  |  |
| None | 142 (11.5%) | 157 (12.7%) | 146 (11.9%) | 234 (18.9%) | 240 (19.4%) | 300 (24.4%) | 338 (27.3%) | 392 (31.7%) | 454 (36.8%) | 2,403 (21.6%) |
| 1—3 days per month | 124 (10.0%) | 97 (7.8%) | 117 (9.5%) | 160 (12.9%) | 133 (10.8%) | 139 (11.3%) | 132 (10.7%) | 124 (10.0%) | 137 (11.1%) | 1,163 (10.5%) |
| 1 day per week | 189 (15.3%) | 171 (13.8%) | 218 (17.7%) | 217 (17.5%) | 229 (18.5%) | 212 (17.2%) | 212 (17.2%) | 161 (13.0%) | 162 (13.1%) | 1,771 (15.9%) |
| > 1 day per week | 691 (55.9%) | 650 (52.5%) | 609 (49.5%) | 534 (43.2%) | 533 (43.1%) | 484 (39.3%) | 444 (35.9%) | 414 (33.5%) | 324 (26.3%) | 4,683 (42.1%) |
| Everyday | 90 (7.3%) | 163 (13.2%) | 140 (11.4%) | 92 (7.4%) | 102 (8.2%) | 97 (7.9%) | 110 (8.9%) | 145 (11.7%) | 156 (12.7%) | 1,095 (9.9%) |
| ***Vigorous Physical Activity*** |  |  |  |  |  |  |  |  |  |  |
| None | 639 (51.7%) | 681 (55.1%) | 678 (55.2%) | 676 (54.8%) | 697 (56.5%) | 716 (58.3%) | 782 (63.5%) | 828 (67.0%) | 897 (73.0%) | 6,594 (59.4%) |
| 1—3 days per month | 116 (9.4%) | 80 (6.5%) | 94 (7.6%) | 94 (7.6%) | 88 (7.1%) | 96 (7.8%) | 98 (8.0%) | 71 (5.7%) | 70 (5.7%) | 807 (7.0%) |
| 1 day per week | 110 (8.9%) | 120 (9.7%) | 121 (9.8%) | 116 (9.4%) | 116 (9.4%) | 110 (9.0%) | 101 (8.2%) | 99 (8.0%) | 70 (5.7%) | 963 (8.7%) |
| > 1 day per week | 340 (27.5%) | 315 (25.5%) | 305 (24.8%) | 322 (26.1%) | 308 (25.0%) | 274 (22.3%) | 227 (18.4%) | 203 (16.4%) | 154 (12.5) | 2,448 (22.4%) |
| Everyday | 32 (2.6%) | 40 (3.2%) | 31 (2.5%) | 25 (2.0%) | 24 (1.9%) | 32 (2.6%) | 23 (1.9%) | 35 (2.8%) | 38 (3.1%) | 280 (2.5%) |
| ***Race*** |  |  |  |  |  |  |  |  |  |  |
| White/Caucasian | 1,056 (85.3%) | 1,056 (85.3%) | 1,056 (85.3%) | 1,056 (85.3%) | 1,056 (85.3%) | 1,056 (85.3%) | 1,056 (85.3%) | 1,056 (85.3%) | 1,056 (85.3%) | 9,504 (85.3%) |
| Black/African American | 154 (12.4%) | 154 (12.4%) | 154 (12.4%) | 154 (12.4%) | 154 (12.4%) | 154 (12.4%) | 154 (12.4%) | 154 (12.4%) | 154 (12.4%) | 1,386 (12.4%) |
| Other | 28 (2.3%) | 28 (2.3%) | 28 (2.3%) | 28 (2.3%) | 28 (2.3%) | 28 (2.3%) | 28 (2.3%) | 28 (2.3%) | 28 (2.3%) | 252 (2.3%) |
| ***Sex*** |  |  |  |  |  |  |  |  |  |  |
| Male | 479 (38.7%) | 479 (38.7%) | 479 (38.7%) | 479 (38.7%) | 479 (38.7%) | 479 (38.7%) | 479 (38.7%) | 479 (38.7%) | 479 (38.7%) | 4,311 (38.7%) |
| Female | 759 (61.3%) | 759 (61.3%) | 759 (61.3%) | 759 (61.3%) | 759 (61.3%) | 759 (61.3%) | 759 (61.3%) | 759 (61.3%) | 759 (61.3%) | 6,831 (61.3%) |
| ***Education Attainment*** |  |  |  |  |  |  |  |  |  |  |
| less than high school | 160 (12.9%) | 160 (12.9%) | 160 (12.9%) | 160 (12.9%) | 160 (12.9%) | 160 (12.9%) | 160 (12.9%) | 160 (12.9%) | 160 (12.9%) | 1,440 (12.9%) |
| GED | 75 (6.1%) | 75 (6.1%) | 75 (6.1%) | 75 (6.1%) | 75 (6.1%) | 75 (6.1%) | 75 (6.1%) | 75 (6.1%) | 75 (6.1%) | 675 (6.1%) |
| High-school graduate | 431 (34.8%) | 431 (34.8%) | 431 (34.8%) | 431 (34.8%) | 431 (34.8%) | 431 (34.8%) | 431 (34.8%) | 431 (34.8%) | 431 (34.8%) | 3,879 (34.8%) |
| Some college | 292 (23.6%) | 292 (23.6%) | 292 (23.6%) | 292 (23.6%) | 292 (23.6%) | 292 (23.6%) | 292 (23.6%) | 292 (23.6%) | 292 (23.6%) | 2,628 (23.6%) |
| College and above | 280 (22.6%) | 280 (22.6%) | 280 (22.6%) | 280 (22.6%) | 280 (22.6%) | 280 (22.6%) | 280 (22.6%) | 280 (22.6%) | 280 (22.6%) | 2,520 (22.6%) |
| ***Marital status*** |  |  |  |  |  |  |  |  |  |  |
| Unmarried | 341 (27.6%) | 341 (27.6%) | 341 (27.6%) | 341 (27.6%) | 341 (27.6%) | 341 (27.6%) | 341 (27.6%) | 341 (27.6%) | 341 (27.6%) | 3,069 (27.6%) |
| Married | 896 (72.4%) | 896 (72.4%) | 896 (72.4%) | 896 (72.4%) | 896 (72.4%) | 896 (72.4%) | 896 (72.4%) | 896 (72.4%) | 896 (72.4%) | 8,064 (72.4%) |
| ***Socioeconomic Status*** |  |  |  |  |  |  |  |  |  |  |
| Poverty class | 69 (5.6%) | 85 (6.9%) | 86 (7.0%) | 106 (8.6%) | 110 (8.9%) | 115 (9.3%) | 115 (9.3%) | 130 (10.5%) | 136 (11.0%) | 952 (8.6%) |
| Lower-middle class | 87 (7.0%) | 75 (6.1%) | 77 (6.3%) | 90 (7.3%) | 80 (6.5%) | 91 (7.4%) | 80 (6.5%) | 83 (6.7%) | 92 (7.4%) | 755 (6.8%) |
| Middle class | 133 (10.7%) | 116 (9.4%) | 110 (8.9%) | 114 (9.2%) | 140 (11.3%) | 105 (8.5%) | 127 (10.3%) | 116 (9.4%) | 109 (8.8%) | 1,070 (9.6%) |
| Upper middle class | 178 (14.4%) | 150 (12.1%) | 163 (13.3%) | 164 (13.2%) | 162 (13.1%) | 175 (14.1%) | 175 (14.1%) | 161 (13.0%) | 147 (11.9%) | 1,475 (13.2%) |
| Wealthy | 437 (35.3%) | 420 (33.9%) | 388 (31.5%) | 393 (31.7%) | 399 (32.2%) | 384 (31.0%) | 370 (29.9%) | 367 (29.6%) | 369 (29.8%) | 3,527 (31.7%) |
| Super wealthy | 334 (27.0%) | 392 (31.7%) | 406 (33.0%) | 371 (30.0%) | 347 (28.0%) | 368 (29.7%) | 371 (30.0%) | 381 (30.8%) | 385 (31.1%) | 3,355 (30.1%) |
|  |  |  |  |  |  |  |  |  |  |  |
| Body Mass Index | 27.767 (5.262) | 28.235 (5.543) | 28.296 (5.610) | 28.201 (5.672) | 28.077 (5.573) | 28.016 (5.644) | 27.801 (5.522) | 27.614 (5.663) | 27.178 (5.699) | 27.909 (5.586) |
| Age | 67.222 (5.951) | 69.206 (5.955) | 71.193 (5.960) | 73.543 (6.015) | 75.300 (5.952) | 77.154 (5.967) | 79.350 (5.984) | 81.303 (5.959) | 83.326 (5.974) | 75.292 (7.910) |
| Drinking status | 1.324 (2.211) | 1.314 (2.243) | 1.283 (2.212) | 1.285 (2.206) | 1.237 (2.166) | 1.218 (2.146) | 1.155 (2.122) | 1.156 (2.136) | 1.061 (2.097) | 1.226 (2.172) |
| Smoking status | 0.093 (0.290) | 0.084 (0.277) | 0.076 (0.265) | 0.062 (0.241) | 0.058 (0.233) | 0.051 (0.220) | 0.047 (0.212) | 0.041 (0.199) | 0.038 (0.192) | 0.061 (0.239) |

Note: Cognitive functioning, age, drinking, body mass index, and smoking all report the means and standard deviations (in parentheses).

Table S8. Complete Sample Characteristics, by multimorbidity count.

|  | | Multimorbidity Count | | | | | | | | | |
| --- | --- | --- | --- | --- | --- | --- | --- | --- | --- | --- | --- |
|  | | 0 | 1 | 2 | 3 | 4 | 5 | 6 | 7 | 8 | Total |
| **N** | | 698 (6.3%) | 2,244 (20.2%) | 3,234 (29.0%) | 2,688 (24.1%) | 1,464 (13.1%) | 569 (5.1%) | 190 (1.7%) | 43 (0.4%) | 4 (0.0%) | 11,134 (100.0%) |
| *Cognition* | | 23.240 (3.884) | 23.197 (4.428) | 23.038 (4.387) | 22.057 (4.571) | 21.235 (4.716) | 21.024 (4.746) | 19.853 (4.006) | 18.474 (4.336) | 23.250 (0.957) | 22.444 (4.549) |
| *Disability* | |  |  |  |  |  |  |  |  |  |  |
| 0 | 1 (0.1%) | | 1 (0.0%) | 4 (0.1%) | 2 (0.1%) | 4 (0.3%) | 3 (0.5%) | 2 (1.1%) | 0 (0.0%) | 0 (0.0%) | 17 (0.2%) |
| 1 | | 0 (0.0%) | 2 (0.1%) | 2 (0.1%) | 3 (0.1%) | 5 (0.3%) | 6 (1.1%) | 4 (2.1%) | 0 (0.0%) | 0 (0.0%) | 22 (0.2%) |
| 2 | | 1 (0.1%) | 2 (0.1%) | 11 (0.3%) | 8 (0.3%) | 5 (0.3%) | 6 (1.1%) | 4 (2.1%) | 2 (4.7%) | 0 (0.0%) | 39 (0.4%) |
| 3 | | 1 (0.1%) | 0 (0.0%) | 10 (0.3%) | 16 (0.6%) | 22 (1.5%) | 19 (3.3%) | 5 (2.6%) | 6 (14.0%) | 0 (0.0%) | 79 (0.7%) |
| 4 | | 4 (0.6%) | 4 (0.2%) | 19 (0.6%) | 24 (0.9%) | 23 (1.6%) | 16 (2.8%) | 10 (5.3%) | 4 (9.3%) | 0 (0.0%) | 104 (0.9%) |
| 5 | | 2 (0.3%) | 10 (0.4%) | 33 (1.0%) | 57 (2.1%) | 57 (3.9%) | 42 (7.4%) | 15 (7.9%) | 2 (4.7%) | 0 (0.0%) | 218 (2.0%) |
| 6 | | 2 (0.3%) | 17 (0.8%) | 57 (1.8%) | 61 (2.3%) | 71 (4.8%) | 36 (6.3%) | 20 (10.5%) | 2 (4.7%) | 2 (50.0%) | 268 (2.4%) |
| 7 | | 5 (0.7%) | 46 (2.1%) | 96 (3.0%) | 132 (4.9%) | 111 (7.6%) | 48 (8.4%) | 20 (10.5%) | 5 (11.6%) | 2 (50.0%) | 465 (4.2%) |
| 8 | | 30 (4.3%) | 139 (6.2%) | 284 (8.8%) | 315 (11.7%) | 247 (16.9%) | 88 (15.5%) | 33 (17.4%) | 7 (16.3%) | 0 (0.0%) | 1,143 (10.3%) |
| 9 | | 652 (93.4%) | 2,022 (90.1%) | 2,718 (84.0%) | 2,070 (77.0%) | 919 (62.8%) | 304 (53.4%) | 76 (40.0%) | 15 (34.9%) | 0 (0.0%) | 8,776 (78.8%) |
| 10 | | 0 (0.0%) | 0 (0.0%) | 0 (0.0%) | 0 (0.0%) | 0 (0.0%) | 1 (0.2%) | 1 (0.5%) | 0 (0.0%) | 0 (0.0%) | 2 (0.0%) |
| *Physical Function* | |  |  |  |  |  |  |  |  |  |  |
| 0 | | 1 (0.1%) | 4 (0.2%) | 16 (0.5%) | 37 (1.4%) | 46 (3.1%) | 25 (4.4%) | 24 (12.6%) | 7 (16.3%) | 1 (25.0%) | 161 (1.4%) |
| 1 | | 1 (0.1%) | 11 (0.5%) | 73 (2.3%) | 98 (3.6%) | 108 (7.4%) | 71 (12.5%) | 37 (19.5%) | 11 (25.6%) | 2 (50.0%) | 412 (3.7%) |
| 2 | | 8 (1.1%) | 38 (1.7%) | 117 (3.6%) | 139 (5.2%) | 113 (7.7%) | 71 (12.5%) | 21 (11.1%) | 10 (23.3%) | 1 (25.0%) | 518 (4.7%) |
| 3 | | 10 (1.4%) | 57 (2.5%) | 183 (5.7%) | 177 (6.6%) | 160 (10.9%) | 86 (15.1%) | 31 (16.3%) | 5 (11.6%) | 0 (0.0%) | 709 (6.4%) |
| 4 | | 14 (2.0%) | 93 (4.1%) | 200 (6.2%) | 230 (8.6%) | 147 (10.0%) | 56 (9.8%) | 18 (9.5%) | 7 (16.3%) | 0 (0.0%) | 765 (6.9%) |
| 5 | | 22 (3.2%) | 120 (5.3%) | 269 (8.3%) | 296 (11.0%) | 185 (12.6%) | 67 (11.8%) | 14 (7.4%) | 1 (2.3%) | 0 (0.0%) | 974 (8.7%) |
| 6 | | 33 (4.7%) | 217 (9.7%) | 349 (10.8%) | 366 (13.6%) | 166 (11.3%) | 63 (11.1%) | 18 (9.5%) | 2 (4.7%) | 0 (0.0%) | 1,214 (10.9%) |
| 7 | | 67 (9.6%) | 278 (12.4%) | 517 (16.0%) | 398 (14.8%) | 181 (12.4%) | 49 (8.6%) | 12 (6.3%) | 0 (0.0%) | 0 (0.0%) | 1,502 (13.5%) |
| 8 | | 133 (19.1%) | 465 (20.7%) | 595 (18.4%) | 425 (15.8%) | 194 (13.3%) | 43 (7.6%) | 10 (5.3%) | 0 (0.0%) | 0 (0.0%) | 1,865 (16.8%) |
| 9 | | 409 (58.6%) | 961 (42.8%) | 915 (28.3%) | 522 (19.4%) | 164 (11.2%) | 38 (6.7%) | 5 (2.6%) | 0 (0.0%) | 0 (0.0%) | 3,014 (27.1%) |
| *Light Activity* | |  |  |  |  |  |  |  |  |  |  |
| None | | 32 (4.6%) | 138 (6.1%) | 250 (7.7%) | 337 (12.6%) | 271 (18.5%) | 125 (22.0%) | 62 (32.6%) | 21 (48.8%) | 4 (100.0%) | 1,240 (11.1%) |
| 1—3 days per month | | 49 (7.0%) | 124 (5.5%) | 221 (6.8%) | 238 (8.9%) | 158 (10.8%) | 55 (9.7%) | 17 (8.9%) | 5 (11.6%) | 0 (0.0%) | 867 (7.8%) |
| 1 day per week | | 163 (23.4%) | 553 (24.6%) | 873 (27.0%) | 790 (29.4%) | 427 (29.2%) | 150 (26.4%) | 45 (23.7%) | 12 (27.9%) | 0 (0.0%) | 3,013 (27.1%) |
| > 1 day per week | | 389 (55.7%) | 1,190 (53.0%) | 1,545 (47.8%) | 1,112 (41.4%) | 522 (35.7%) | 203 (35.7%) | 57 (30.0%) | 2 (4.7%) | 0 (0.0%) | 5,020 (45.1%) |
| Everyday | | 65 (9.3%) | 239 (10.7%) | 341 (10.6%) | 206 (7.7%) | 85 (5.8%) | 35 (6.2%) | 9 (4.7%) | 3 (7.0%) | 0 (0.0%) | 983 (8.8%) |
| *Moderate Activity* | |  |  |  |  |  |  |  |  |  |  |
| None | | 74 (10.6%) | 262 (11.7%) | 568 (17.6%) | 658 (24.5%) | 491 (33.6%) | 227 (39.9%) | 98 (51.6%) | 23 (53.5%) | 2 (50.0%) | 2,403 (21.6%) |
| 1—3 days per month | | 48 (6.9%) | 198 (8.8%) | 328 (10.2%) | 333 (12.4%) | 183 (12.5%) | 57 (10.0%) | 13 (6.8%) | 3 (7.0%) | 0 (0.0%) | 1,163 (10.5%) |
| 1 day per week | | 110 (15.8%) | 319 (14.2%) | 575 (17.8%) | 437 (16.3%) | 222 (15.2%) | 74 (13.0%) | 27 (14.2%) | 6 (14.0%) | 1 (25.0%) | 1,771 (15.9%) |
| > 1 day per week | | 379 (54.4%) | 1,218 (54.4%) | 1,395 (43.2%) | 1,024 (38.2%) | 451 (30.8%) | 167 (29.3%) | 41 (21.6%) | 7 (16.3%) | 1 (25.0%) | 4,683 (42.1%) |
| Everyday | | 86 (12.3%) | 244 (10.9%) | 361 (11.2%) | 230 (8.6%) | 115 (7.9%) | 44 (7.7%) | 11 (5.8%) | 4 (9.3%) | 0 (0.0%) | 1,095 (9.9%) |
| *Vigorous Activity* | |  |  |  |  |  |  |  |  |  |  |
| None | | 321 (46.1%) | 1,022 (45.7%) | 1,646 (51.1%) | 1,442 (53.9%) | 851 (58.2%) | 315 (55.5%) | 109 (57.7%) | 26 (60.5%) | 3 (75.0%) | 5,735 (51.7%) |
| 1—3 days per month | | 64 (9.2%) | 180 (8.1%) | 286 (8.9%) | 217 (8.1%) | 102 (7.0%) | 37 (6.5%) | 5 (2.6%) | 0 (0.0%) | 0 (0.0%) | 891 (8.0%) |
| 1 day per week | | 68 (9.8%) | 250 (11.2%) | 292 (9.1%) | 235 (8.8%) | 77 (5.3%) | 31 (5.5%) | 6 (3.2%) | 4 (9.3%) | 0 (0.0%) | 963 (8.7%) |
| > 1 day per week | | 203 (29.1%) | 654 (29.3%) | 741 (23.0%) | 469 (17.5%) | 203 (13.9%) | 72 (12.7%) | 21 (11.1%) | 1 (2.3%) | 0 (0.0%) | 2,364 (21.3%) |
| Everyday | | 41 (5.9%) | 129 (5.8%) | 257 (8.0%) | 310 (11.6%) | 228 (15.6%) | 113 (19.9%) | 48 (25.4%) | 12 (27.9%) | 1 (25.0%) | 1,139 (10.3%) |
| Age | | 72.586 (7.921) | 73.410 (7.491) | 75.050 (7.622) | 76.402 (7.939) | 77.430 (8.045) | 76.359 (8.019) | 76.763 (7.946) | 73.302 (6.909) | 69.000 (2.582) | 75.292 (7.910) |
| Marital Status | | 0.716 (0.451) | 0.776 (0.417) | 0.731 (0.443) | 0.720 (0.449) | 0.696 (0.460) | 0.650 (0.477) | 0.574 (0.496) | 0.581 (0.499) | 0.000 (0.000) | 0.724 (0.447) |
| Socioeconomic Status | | 3.802 (1.418) | 3.850 (1.316) | 3.599 (1.484) | 3.330 (1.561) | 2.966 (1.726) | 2.583 (1.720) | 2.195 (1.823) | 2.093 (1.998) | 0.250 (0.500) | 3.431 (1.573) |
| Body Mass Index | | 25.673 (4.641) | 26.038 (4.450) | 27.943 (5.242) | 28.798 (5.914) | 28.962 (5.833) | 29.119 (5.960) | 30.724 (6.689) | 37.153 (9.962) | 46.325 (3.909) | 27.909 (5.586) |
| Drinking Status | | 1.670 (2.377) | 1.734 (2.447) | 1.226 (2.189) | 1.094 (2.048) | 0.798 (1.822) | 0.701 (1.755) | 0.571 (1.554) | 0.186 (0.588) | 0.000 (0.000) | 1.226 (2.172) |
| Smoking Status | | 0.096 (0.295) | 0.054 (0.225) | 0.065 (0.247) | 0.040 (0.195) | 0.070 (0.255) | 0.095 (0.293) | 0.084 (0.278) | 0.047 (0.213) | 0.000 (0.000) | 0.061 (0.239) |
